# *Lactobacillus reuteri*-derived extracellular vesicles maintain intestinal immune homeostasis against lipopolysaccharide-induced inflammatory responses in broilers

**DOI:** 10.1186/s40104-020-00532-4

**Published:** 2021-02-17

**Authors:** Rujiu Hu, Hua Lin, Mimi Wang, Yuezhen Zhao, Haojing Liu, Yuna Min, Xiaojun Yang, Yupeng Gao, Mingming Yang

**Affiliations:** College of Animal Science and Technology, Northwest A&F University, Yangling, 712100 Shaanxi China

**Keywords:** Chickens, Extracellular vesicles, Immune responses, Inflammation, *Lactobacillu*s, Microbiota-host communication, Probiotics

## Abstract

**Background:**

*Lactobacillus reuteri* strains are widely used as probiotics to prevent and treat inflammatory bowel disease by modulating the host’s immune system. However, the underlying mechanisms by which they communicate with the host have not been clearly understood. Bacterial extracellular vesicles (EVs) have been considered as important mediators of host-pathogen interactions, but their potential role in commensals-host crosstalk has not been widely studied. Here, we investigated the regulatory actions of EVs produced by *L. reuteri* BBC3, a gut-associated commensal bacterium of Black-Bone chicken, in the development of lipopolysaccharide (LPS)-induced intestinal inflammation in a chicken model using both *in vivo* and *in vitro* experiments.

**Results:**

*L. reuteri* BBC3 produced nano-scale membrane vesicles with the size range of 60–250 nm. Biochemical and proteomic analyses showed that *L. reuteri* BBC3-derived EVs (LrEVs) carried DNA, RNA and several bioactive proteins previously described as mediators of other probiotics’ beneficial effects such as glucosyltransferase, serine protease and elongation factor Tu. *In vivo* broiler experiments showed that administration of LrEVs exerted similar effects as *L. reuteri* BBC3 in attenuating LPS-induced inflammation by improving growth performance, reducing mortality and decreasing intestinal injury. LrEVs suppressed the LPS-induced expression of pro-inflammatory genes (*TNF-α*, *IL-1β*, *IL-6*, *IL-17* and *IL-8*), and improved the expression of anti-inflammatory genes (*IL-10* and *TGF-β*) in the jejunum. LrEVs could be internalized by chicken macrophages. *In vitro* pretreatment with LrEVs reduced the gene expression of *TNF-α*, *IL-1β* and *IL-6* by suppressing the NF-κB activity, and enhanced the gene expression of *IL-10* and *TGF-β* in LPS-activated chicken macrophages. Additionally, LrEVs could inhibit Th1- and Th17-mediated inflammatory responses and enhance the immunoregulatory cells-mediated immunosuppression in splenic lymphocytes of LPS-challenged chickens through the activation of macrophages. Finally, we revealed that the reduced content of both vesicular proteins and nucleic acids attenuated the suppression of LrEVs on LPS-induced inflammatory responses in *ex vivo* experiments, suggesting that they are essential for the LrEVs-mediated immunoregulation.

**Conclusions:**

We revealed that LrEVs participated in maintaining intestinal immune homeostasis against LPS-induced inflammatory responses in a chicken model. Our findings provide mechanistic insight into how commensal and probiotic *Lactobacillu*s species modulate the host’s immune system in pathogens-induced inflammation.

**Supplementary Information:**

The online version contains supplementary material available at 10.1186/s40104-020-00532-4.

## Background

Membrane vesicles (MVs) have been recognized as a form of cell-cell communication used by almost all domains of life: bacteria, archaea and eukaryotes [1]. Over the years, they have been largely ignored in the field of microbiology. It is only in the last decade that advances in biochemical analysis have led researchers to begin to elucidate the biogenesis and functions of MVs [2, 3]. Bacterial MVs are released from the cell surface into the extracellular environment, and thus also referred to as extracellular vesicles (EVs). These vesicles are composed of spherical proteolipids with a diameter ranging from 50 to 500 nm [1]. Bacterial EVs contain various bioactive molecules of the parental bacteria, including proteins, lipids, nucleic acids and polysaccharides, which are involved in a large number of pathological and physiological functions in intercellular interactions such as nutrient acquisition, biofilm formation, stress response, delivery of toxins and virulence factors and invasion of host and immune regulation [2, 3]. Gram-negative bacterial EVs are mainly derived from the outer membrane of the cell envelope, and thus also termed outer membrane vesicles (OMVs) [4]. Most of the early work regarding bacterial EVs was conducted mainly on Gram-negative bacteria, especially pathogenic bacteria, which has shown that the EVs can mediate bacterial pathogenesis and invasion by delivering toxins and virulence factors into the host cells [4]. OMVs can also activate the innate and adaptive immune responses and induce the protective immunity similar to that induced by the intact bacteria *in vivo*, suggesting that these OMVs can be used for the development of novel vaccine candidates and adjuvants [5, 6].

Few studies, however, have been performed on Gram-positive bacterial EVs, because it is believed to be difficult for Gram-positive bacteria to release EVs due to the presence of a thick cell wall [1]. It was not until 2009 when direct evidence was provided for the existence of EVs in Gram-positive bacteria by transmission electron microscopy and proteomic analyses of EVs from *Staphylococcus aureus* [7]. Since then, more and more Gram-positive bacteria have been demonstrated to be able to produce EVs, such as *Bacillus anthracis* [8], *Streptococcus pneumoniae* [9], *Bacillus subtilis* [10] and *Clostridium perfringens* [11]. As the most common Gram-positive bacteria, several *Lactobacillus* species have been recently discovered to produce EVs. Rubio et al. [12] demonstrated the probiotic strain *L. casei* BL23 produced biofunctional EVs. Li et al. [13] showed that *L. plantarum* WCFS1 secreted EVs with an average size of 101 nm. EVs derived from *L. acidophilus* and *L. paracasei* have also been isolated and characterized [14, 15]. Recently, several studies also demonstrated the existence of EVs in *L. reuteri* strains [14, 16]. However, the potential roles and functions of these *Lactobacillus*-derived vesicles have not been extensively studied.

With the gradual ban on the usage of antibiotic growth promoters in today’s intensive broiler industry worldwide, probiotics have been widely used as alternatives to prevent and treat various inflammatory disorders in the gastrointestinal tract. *L. reuteri* species have been demonstrated to have beneficial attributes, particularly the ability to modulating the development and function of the host’s immune system, in humans and various animals, including rodents, pigs, turkeys and chickens [17, 18]. A previous study has demonstrated that *L. reuteri* inhibits the secretion of pro-inflammatory cytokines tumour necrosis factor (TNF)-α and interleukin (IL)-6 in murine dendritic cells (DCs) [19]. Lin et al. [20] revealed that *L. reuteri* exerted the suppressive effect on the production of TNF-α in lipopolysaccharide (LPS)-challenged human macrophages (Mφ). *L. reuteri* has also been shown to have an ability to induce the development of regulatory T cells (Tregs) that inhibit inflammatory responses [21, 22]. In chickens, *L. reuteri* is one of the most abundant *Lactobacillus* species in the intestine and has been shown to have an anti-inflammatory effect against pathogens [23–25]. However, the underlying molecular mechanisms of *L. reuteri*-host interaction have not been clearly understood.

Based on these findings, the present study was initiated to explore whether EVs derived from the probiotic strain *L. reuteri* BBC3 (LrEVs), a gut-associated commensal bacterium of Black-Bone chicken, mediate immunoregulation in the chicken intestine, especially anti-inflammatory actions. To achieve this aim, we characterized the protein composition of LrEVs using proteomic analysis, evaluated the protective effects of LrEVs in a chicken model of LPS-induced intestinal inflammation and investigated the immune responses mediated by LrEVs in the LPS-activated Mφ model and Mφ-splenic lymphocytes coculture system *in vitro*. We further attempted to understand the roles of vesicular proteins and nucleic acids in LrEVs-mediated immunomodulation using an *ex vivo* experiment.

## Materials and methods

### Bacterial strain and culture conditions

*L. reuteri* BBC3 (GenBank accession: MT476913) was previously isolated by our laboratory from the gastrointestinal tract of healthy Black-Bone chickens in free-range farms (Lueyang, Shaanxi Province, China). This strain was identified by morphological and physicochemical characterizations and 16S rRNA sequence analysis. *L. reuteri* BBC3 was routinely incubated in MRS broth at 37 °C under anaerobic conditions.

### Isolation and purification of LrEVs

LrEVs were isolated from the culture supernatants of *L. reuteri* BBC3 using a series of (ultra)centrifugation steps as described previously [26, 27]. Briefly, after growing in MRS broth for 16 h, the bacteria-free culture supernatants were collected by centrifugation (12,000×*g*, 20 min, 4 °C), filtered with a 0.45-μm bottle top vacuum filter (Corning), and then concentrated using an Amicon Ultrafiltration system (Millipore) with a 100-kDa filter. The LrEVs pellets were obtained by ultracentrifugation (150,000×*g*, 2 h, 4 °C), washed in sterile phosphate buffer saline (PBS; pH 7.4) and then purified by density gradient centrifugation. For purification, LrEVs were covered with discontinuous Optiprep (Sigma, #D1556) step-gradient ranging from 10% to 55% (w/v) and subjected to ultracentrifugation (16 h, 180,000×*g*, 4 °C). After centrifugation, nanoparticle tracking analysis (NTA) was performed to detect the particle numbers of the resulting fractions. These vesicle-rich fractions were pooled and ultra-centrifuged to remove OptiPrep. The purified LrVs were subjected to filtration (0.45 μm, Millipore) to eliminate the potential bacterial contamination and stored at − 80 °C until future use.

### Electron microscopy and nanoparticle tracking analysis

Morphological characteristics of *L. reuteri* BBC3 and LrEVs were detected by scanning electron microscopy using a Field Emission Scanning Electron Microscope (S-4800, Hitachi, Tokyo, Japan). The LrEVs were also viewed by transmission electron microscopy using JEM1011 Electron Microscope at 100 kV (JEOL, Tokyo, Japan) as described previously [28]. NTA was performed to detect the diameter and particle number of the LrEVs using an NS300 nanoparticle analyzer (Malvern, Worchestershire, UK) with the operating parameters as described in our previous study [27].

### Biochemical analysis

The protein content of the LrEVs was determined by the TaKaRa BCA Protein Assay Kit (TaKaRa Bio, Beijing, China; #T9300A) following the manufacturer’s instructions. DNA and RNA in the LrEVs were quantified by using the Quant-iT™ dsDNA Assay Kit (Invitrogen, #Q33130) and Quant-iT™ RNA Assay Kit (Invitrogen, #Q33140) following the manufacturer’s instructions, respectively. The contents of protein, DNA and RNA were normalized by using 1 × 10^11^ particles of vesicles. The amount of LrEVs used in the following experiments was based on the protein content.

### Proteomic analysis

Triplicate biological LrEVs samples were sent to Hangzhou PTM Biolabs (Hangzhou, Zhejiang Province, China) for proteomic analysis. In brief, the LrEVs samples were lysed by sonication on ice in lysis buffer (8 mol/L urea, 1% protease inhibitor cocktail, 2 mmol/L EDTA), and the protein supernatants were harvested by centrifugation (12,000×*g*, 15 min, 4 °C). The extracted protein solutions were digested with trypsin (Promega, #V5111) at a 1:50 (w/w; trypsin to protein) overnight at 37 °C. After fractionating by the high pH reverse-phase HPLC, the tryptic peptides were processed by tandem mass spectrometry (MS/MS) in Q ExactiveTM Plus (Thermo) coupled online to the UPLC (LC-MS/MS). The resulting spectrum was analyzed against *L. reuteri* genome draft sequence in the UniProt database. The subcellular localization of the identified proteins was predicted by the CELLO v.2.5 online tool (set for Gram-positive bacteria; http://cello.life.nctu.edu.tw/). The biological function of the identified proteins was classified by Gene Ontology (GO) annotation from the UniProt-GOA database (http://www.ebi.ac.uk/GOA/). If some identified proteins were not annotated by UniProt-GOA database, the InterProScan soft (http://www.ebi.ac.uk/interpro/) would be used to annotate protein’s GO function based on the protein sequence alignment method. All parameter settings and bioinformatic annotations were performed as described in our previous study [27].

### Animals and housing

Newly hatched broiler chicks (Arbor Acres) were obtained from Dacheng Poultry Industry Company (Xianyang, Shaanxi Province, China). The chicks were housed in stainless-steel cages in a sterilized room with filtered air, strict sanitary conditions and age-appropriate temperatures. All chicks were fed an age-appropriate commercial diet containing no antibiotic additives. Drinking water and diets were offered ad libitum. All procedures of animal experiments were approved by the Ethics Committee of Animal Care and Use at Northwest A&F University.

### LPS-induced intestinal inflammation in broiler chicken model

A total of 144 broiler chicks (1 male:1 female) were randomly selected and grouped by body weight into 4 treatment groups when they were 7 days of age including: (1) a negative control challenged with PBS (group PBS; average BW = 136.3 ± 3.5 g); (2) a positive control challenged with LPS (group LPS; average BW = 137.1 ± 3.1 g); (3) a group treated with *L. reuteri* BBC3 and challenged with LPS (group LR + LPS; average BW = 136.8 ± 2.8 g); (4) a group treated with LrEVs and challenged with LPS (group LrEVs+LPS; average BW = 135.9 ± 3.4 g). All birds were placed in two-level wired cages and each group had 6 cages (replicates; 3 male and 3 female) of 6 birds each. Three replicates per treatment group were distributed in the upper cages and the other three replicates per treatment group were distributed in the lower cages. At 12, 14 and 18 days of age, the birds in the three LPS-challenged groups were intraperitoneally injected LPS (500 μg/bird; *E. coli* O111:B4 origin; Sigma, #L4391) in 100 μL PBS; birds in the PBS group were intraperitoneally injected an equal volume of sterile PBS. The dosage of LPS challenge was determined based on previous reports [29]. Every other day from 7 to 21 days of age, the birds in the LR + LPS and LrEVs+LPS groups were given by gavage the cultured *L. reuteri* BBC3 (5 × 10^9^ CFU/bird) and purified LrEVs (200 μg/bird) in 200 μL protectant (5% skim milk), respectively; the birds in the remaining two groups were given by gavage an equal volume of PBS in protectant. The chicks were sacrificed by jugular exsanguination after intravenous injection of pentobarbital sodium (20 mg/kg body weight) on day 21 for sample collection.

### Growth performance and intestinal morphology analysis

The body weight, feed intake and the number of deaths were recorded on a replicate basis and used to calculate the growth performance of birds from 7 to 21 days of age, including average daily weight gain (ADWG), average daily feed intake (ADFI), feed gain ratio (F/G). ADWG, ADFI and F/G were adjusted when any bird died. Jejunum tissues were sampled at 21 days of age and fixed in neutral-buffered 4% paraformaldehyde solution. The fixed tissues were dehydrated through a graded series of ethanol (50%, 70%, 85%, 95% and 100% alcohol), embedded into paraffin and sectioned at a thickness of 3–5 μm. After staining with hematoxylin and eosin, the sections were examined with an Olympus BX53F microscope (Olympus, Tokyo, Japan) at 20× magnification. Villus height and crypt depth were measured from ten representative well-preserved villi per segment, then the ratio of villus height to crypt depth (VH/CD) was calculated.

### *In vitro* macrophage assay

HD11 cells, a transformed chicken Mφ cell line derived from bone marrow [30], were used in this study and cultured in the complete PRMI-1640 medium (Gibco, #22400089) supplemented with 10% heat-inactivated fetal bovine serum (FBS; Zeta-Life, #Z7181FBS-500), 100 U/mL penicillin and 100 μg/mL streptomycin (Sigma, #P4333) in an atmosphere of 5% CO^2^ at 37 °C. Visualization of the internalization of LrEVs by HD11 cells was performed as presented in our previous study [27]. Briefly, HD11 cells (5 × 10^5^ cells/mL) were co-incubated with DiI (Sigma, #42364)-labeled LrEVs (10 μg/mL) for 6 h. After washing and staining with 4′, move to the same line (DAPI; Sigma, #D9542), the cells were detected using the high-speed spinning-disk confocal microscope (Andor Revolution XD, Andor Technology, UK).

For the LPS-challenged Mφ assay, HD11 cells (5 × 10^5^ cells/mL), were pretreated with PBS or LrEVs (10 μg/mL) for 12 h and stimulated with PBS or LPS (1 μg/mL) for 12 h. The cells were collected for the determination of cytokine gene expression, NF-κB p65 transcription factor activity and cell viability. Three independent experiments were performed per treatment.

### *In vitro* Mφ-splenic lymphocyte coculture

Splenic lymphocytes were isolated from the spleen of chickens in the LPS group at day 21 using the Chicken Splenic Lymphocyte Isolation Kit (Solarbio, #P9120) according to the manufacturer’s instructions. The isolated splenic lymphocytes were maintained in complete PRMI-1640 medium supplemented with 10% FBS, 100 U/mL penicillin and 100 μg/mL streptomycin in an atmosphere of 5% CO^2^ at 37 °C. For transwell culture, LrEVs- or PBS-pretreated HD11 cells (10 μg/mL LrEVs, 5 × 10^5^ cells/mL, 12 h) were added to the basolateral compartment of a 6-well plate, and splenic lymphocytes (5 × 10^5^ cells/mL) were seeded on the apical compartment of 6-well Hanging Inserts (0.4 μm; Corning, #M150865). After 12-h coculture, splenic lymphocytes were collected to determine the cytokine gene expression. Three independent experiments were performed per treatment.

### Enzymatic treatment of LrEVs

To remove vesicular nucleic acids and proteins, 1 mg/mL of native LrEVs were treated with 10 U/mL of DNase I (Thermo Scientific, #EN0521), 10 U/mL of RNase I (Solarbio, #R8021), and 1 mg/mL of proteinase K-agarose (Sigma, #P9290), respectively, as described previously [16, 31]. The native LrEVs were incubated with DNase I and RNase I at 37 °C for 30 min, and with proteinase K-agarose at 37 °C for 2 h. The enzymes were inactivated at 75 °C for 1 h. Proteinase K-agarose was additionally removed by centrifugation (12,000×*g*, 1 min).

### *Ex vivo* jejunum explant culture

The culture of jejunum tissues was performed as described previously [32]. Briefly, jejunum explants (5 mm × 5 mm) without lesions from healthy broilers of 21 days old were rinsed under sterile saline solution at 4 °C and maintained in 24-well culture plates with the complete PRMI-1640 medium supplemented with 10% FBS, 0.25 μg/mL fungizone (Sigma-Aldrich, #V900919), 100 U/mL penicillin and 100 μg/mL streptomycin (in an atmosphere of 5% CO^2^ at 37 °C). The explants were pretreated with native LrEVs (10 μg/mL), DNase I and RNase I-treated LrEVs (DR-LrEVs; 10 μg/mL) or proteinase K-agarose-treated LrEVs (PK-LrEVs; 10 μg/mL before proteinase K-agarose treatment) for 6 h and then exposed to LPS (1 μg/mL) for 6 h. The explants were collected to determine cytokine gene expression and myeloperoxidase (MPO) activity. Three independent experiments were performed per treatment.

### Quantitative real-time PCR (qRT-PCR)

Total RNA was extracted from jejunum tissues, HD11 cells, splenic lymphocytes and jejunum explants using the Total RNA Kit (Omega Bio-Tek, #R1034) following the manufacturer’s instructions. After examination of RNA purity and quality, qRT-PCR analysis was conducted with One Step SYBR® PrimeScript™ PLUS RT-PCR Kit (TaKaRa Bio, Beijing, China; #RR096A) in a Real-Time PCR Detection System (CFX96 Touch, Bio-Rad) according to the manufacturer’s instructions. The primer sequences of target genes and a reference gene (β-actin) used for qPCR are shown in Supplementary Table S1. Triplicate qRT-PCR reactions for each sample were conducted under the following settings: 95 °C for 1 min, 40 cycles of 95 °C for 15 s and 60 °C for 30 s. Relative mRNA expression was calculated by the method of the 2^−ΔΔCt^ as described previously [33], and expressed as the fold-change relative to the control, which was normalized to 1.

### NF-κB p65 activity assay

The NF-κB p65 transcription factor activity in the cell nucleus from chicken Mφ was measured using the NF-κB p65 Transcription Factor Assay Kit (Abcam, #ab133112) following the manufacturer’s instructions with some modifications. Briefly, nuclear protein extracts were prepared from cultured Mφ using the Nuclear Extraction Kit (Abcam, #ab113474) according to the manufacturer’s instructions. The extracted nuclear fractions were added into black 96-well plates containing the consensus binding sequence for NF-κB p65 and incubated for 1 h at room temperature. After washing, a chicken reactive rabbit anti-NF-κB p65 antibody (Abcam, #ab16502) was loaded into each well and incubated for 1 h at room temperature. After washing, the HRP-conjugated secondary goat anti-rabbit antibody was added into each well and incubated for 1 h at room temperature. Each well was washed 5 times and loaded with a developing solution and then incubated for 15–45 min at room temperature. The OD_450_ values were measured using a Microplate Reader (Epoch 2, Biotek) following the addition of stop solution to each well. Each sample was determined in triplicate.

### Cell viability

The viabilities of HD11 cells were determined by Trypan Blue dye (Sigma, #T6146) exclusion assay as described previously [34]. Data were presented as the proportion (%) of viable cells (cells excluding blue dye) to the total counted cells.

### MPO activity assay

Jejunum tissues or explants were collected to determine the MPO activity using the Myeloperoxidase Assay Kit (Nanjing Jiancheng Bioengineering Institute, Jiangsu, China; #A044–1-1) following the manufacturer’s instructions. Data were presented as a unit of MPO activity (U) per mg of protein. One U is defined as the amount of enzyme that catalyzes 1 μmol peroxide per minute at 37 °C.

### Statistical analysis

All data were expressed as mean ± standard error of the mean (SEM). Student’s *t*-test was used for the analysis of differences between the two groups. One-way ANOVA with Newman-Keuls test as the post hoc test was performed for comparisons among greater than two groups. Statistical significance was declared at *P* < 0.05. All analyses were performed using Graph Pad Prism software 5.0 (San Diego, CA, USA).

## Results

### *L. reuteri* BBC3 releases nanosized extracellular vesicles

**Fig. 1:**
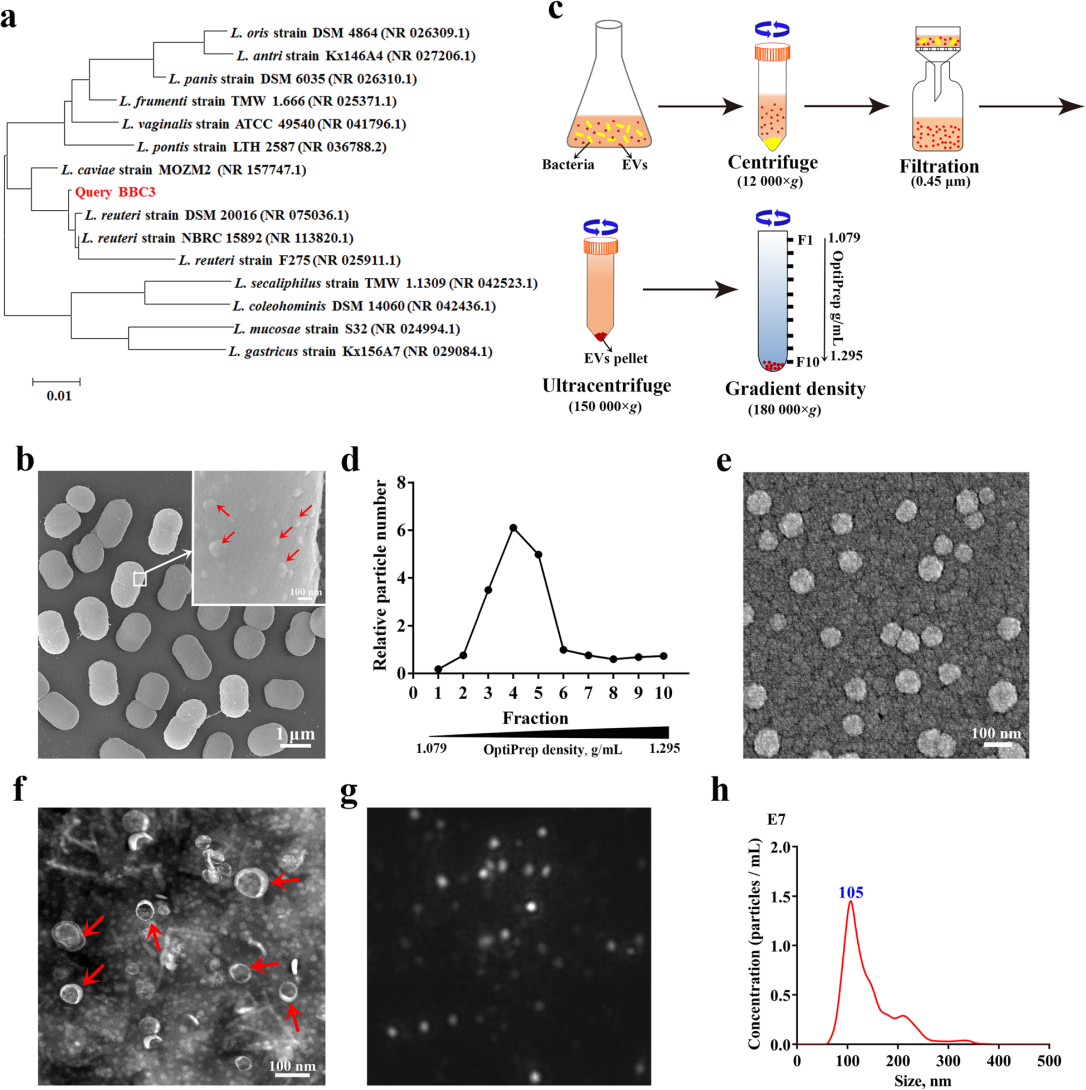
Preparation and characterization of *L. reuteri* BBC3-derived EVs (LrEVs). **a** Phylogenetic diagram of *L. reuteri* BBC3 based on 16S rRNA sequences. The 15 most homologous sequences in the GenBank database were selected for the construction of a phylogenetic tree. **b** Representative image of the scanning electron microscope for *L. reuteri* BBC3 cells showing membrane vesicles on the bacterial cell surface. **c** Isolation and purification procedures of bacterial EVs. **d** LrEVs were purified by the discontinuous density ultracentrifugation. Nanoparticle tracking analysis (NTA) was performed to detect the particle numbers of each gradient fraction. Representative images of the scanning electron microscope (**e)** and transmission electron microscopy (**f**) for LrEVs. **g** Representative image from the recorded movies using a SCMOS camera of Malven NTA 3.0 when LrEVs were characterized by NTA. **h** Concentration and size distribution of the purified LrEVs determined by NTA

The 16S rRNA sequence-based phylogenetic analysis indicated that the *Lactobacillus* isolate used in this study belonged to a subclade of *L. reuteri*, showing 99.2–99.5% similarity to other strains within *L. reuteri* (Fig. 1a). Ultrastructural analysis of the isolate, *L. reuteri* BBC3, showed that single vesicles, occasionally multiple vesicles, shed from the surface of individual bacterial cells (Fig. 1b). As shown in Fig. 3c, these vesicles were obtained from the culture supernatants of *L. reuteri* BBC3 using a series of filtration and centrifugation steps. A large number of vesicle particles were found in fractions 3–5 following purification by density gradient centrifugation (Fig. 1d). The density range for these fractions was from 1.127 to 1.199 g/mL. These EV-containing fractions were pooled and subsequently visualized by scanning electron microscopy (Fig. 1e) and transmission electron microscopy (Fig. 1f). These micrographs revealed that the purified LrEVs were membrane-enclosed structures with spherical morphology, and the majority of these vesicles ranged from 50 nm to 150 nm in diameter. These findings are consistent with the typical results characterized by NTA analysis (Fig. 1g and f), which showed that the sizes of LrEVs ranged from 60 to 250 nm and peaked at 105 nm. Together, these results demonstrate that this *L. reuteri* BBC3 isolate can release nano-sized vesicles.

### Proteomic analysis of LrEVs

**Fig. 2:**
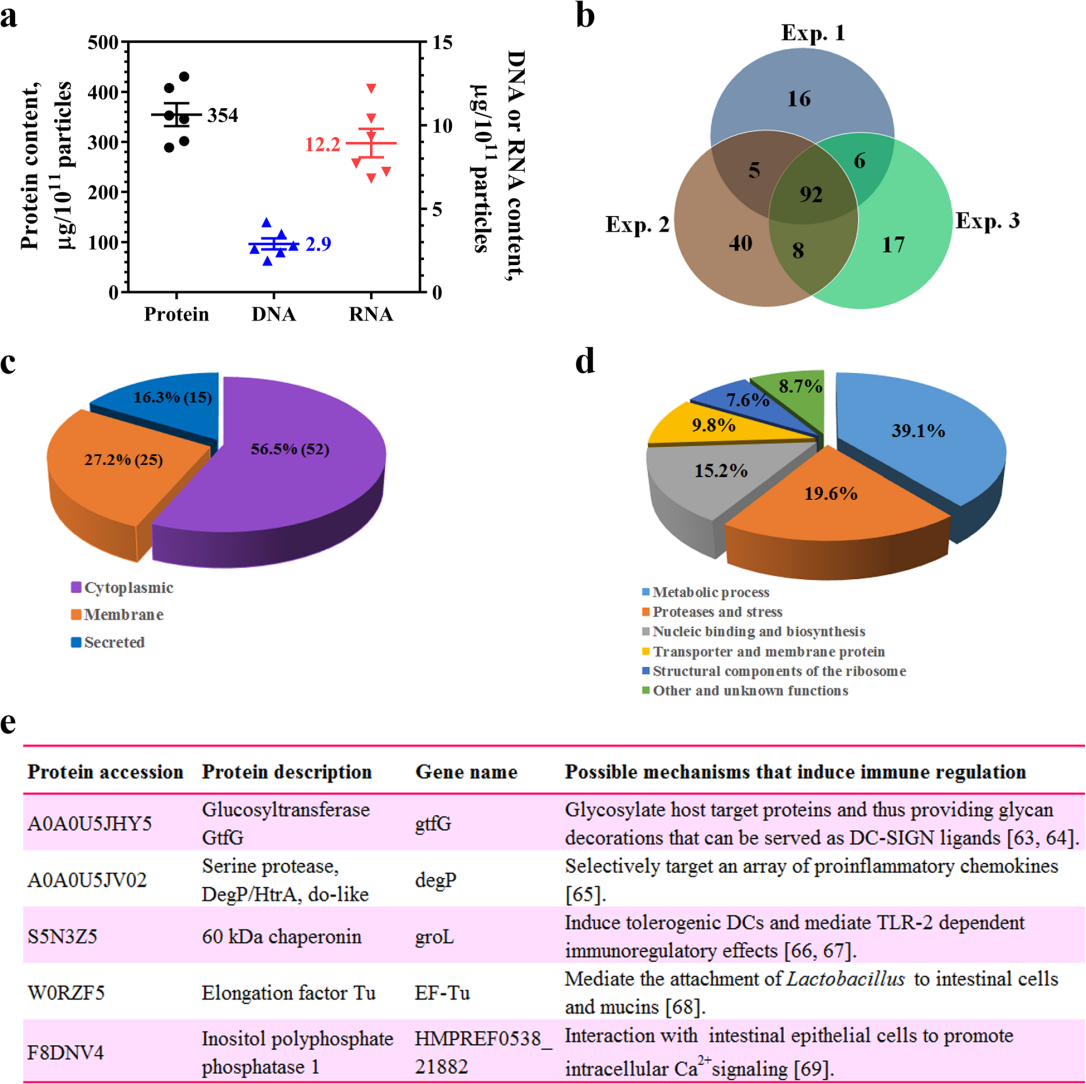
Biochemical and proteomic analyses of LrEVs. **a** Quantifications of protein, DNA and RNA in the LrEVs. **b** Venn diagram showed that 92 overlapping proteins were identified in triplicate samples. **c** Subcellular localization of the identified proteins present in the LrEVs. It was found that 56.5% was cytoplasmic and 43.5% belonged to the membrane and secreted proteins. **d** Biological function classification of the identified proteins. It was found that the majority was either metabolic process (39.1%) or proteases and stress (19.6%). **e** A selected list of the identified proteins which may function in immune regulation, including protein accession, protein description and possible mechanisms

Biochemical analysis revealed that the LrEVs contained DNA, RNA and proteins, and the protein content was noticeably higher than that of DNA or RNA (Fig. 2a). These findings are in accordance with the previously described composition of EVs derived from other *Lactobacillus* strains [12, 14]. Proteomic analysis was further performed to detect the protein profile of LrEVs. A total of 92 overlapping proteins were identified in triplicate biological LrEVs samples and selected for further analysis (Fig. 2b). The subcellular localization of these identified proteins showed that 56.5% was originated from the cytoplasm and 43.5% belonged to membrane proteins and secreted proteins (Fig. 2c). According to the distribution of biological functions analyzed by GO annotation, most of these proteins were classified into metabolic enzymes, proteases, nucleic-binding proteins, transporter and membrane proteins and structural components of the ribosome, suggesting that LrEVs may be involved in metabolism, transporter activity, translation and transcription, signaling and stress, etc. (Fig. 2d). The detailed information of all identified proteins is listed in Supplementary Table S2. Notably, several homologous proteins that were previously described as mediators of anti-inflammatory or beneficial effects in other probiotics or commensal bacterium were also observed in LrEVs, such as glucosyltransferase, serine protease, 60 kDa chaperonin, elongation factor Tu and inositol polyphosphate phosphatase 1 (Fig. 2e). Altogether, these findings reveal that LrEVs carry DNA, RNA and some immunoregulatory proteins, leading us to investigate the functions of these vesicles in the underlying mechanism of bacterium-host interactions.

### *L. reuteri* BBC3 and its EVs attenuate LPS-induced intestinal injury in chickens

**Fig. 3:**
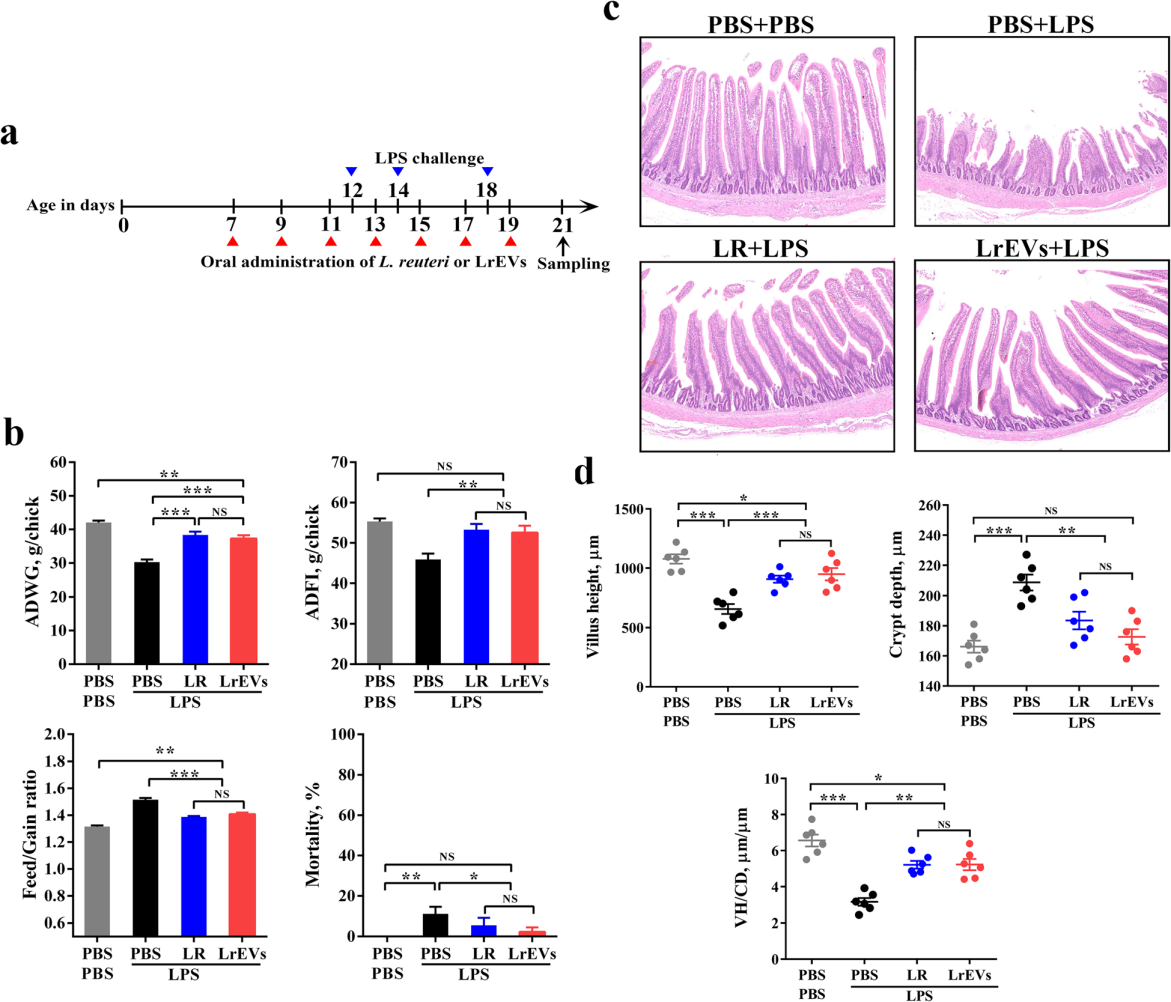
*L. reuteri* BBC3 and LrEVs attenuate lipopolysaccharide (LPS)-induced intestinal injury and inflammation in broiler chickens. **a** Experimental schedule for LPS challenge and administration of *L. reuteri* BBC3 and LrEVs. LPS (500 μg/bird) from *E. coli* O111: B4 was intraperitoneally injected 3 times; *L. reuteri* BBC3 (5 × 10^9^ CFU/bird) and purified LrEVs (200 μg/bird) in 200 μL protectant (5% skim milk) were given 7 times by gavage. **b** Growth performance of each treatment from 7 to 21 days of age, including average daily weight gain (ADWG), average daily feed intake (ADFI), feed gain ratio (F/G) and mortality. **c** Representative image of each treatment group from hematoxylin and eosin-stained jejunum slides. **d** Intestinal morphology analysis based on measurements of villus height, crypt depth and the ratio of villus height to crypt depth (VH/CD) in jejunum tissues. Values are expressed as means ± SEM (*n* = 6). *P < 0.05; **P < 0.01; ***P < 0.001; NS, not significant

**Fig. 4:**
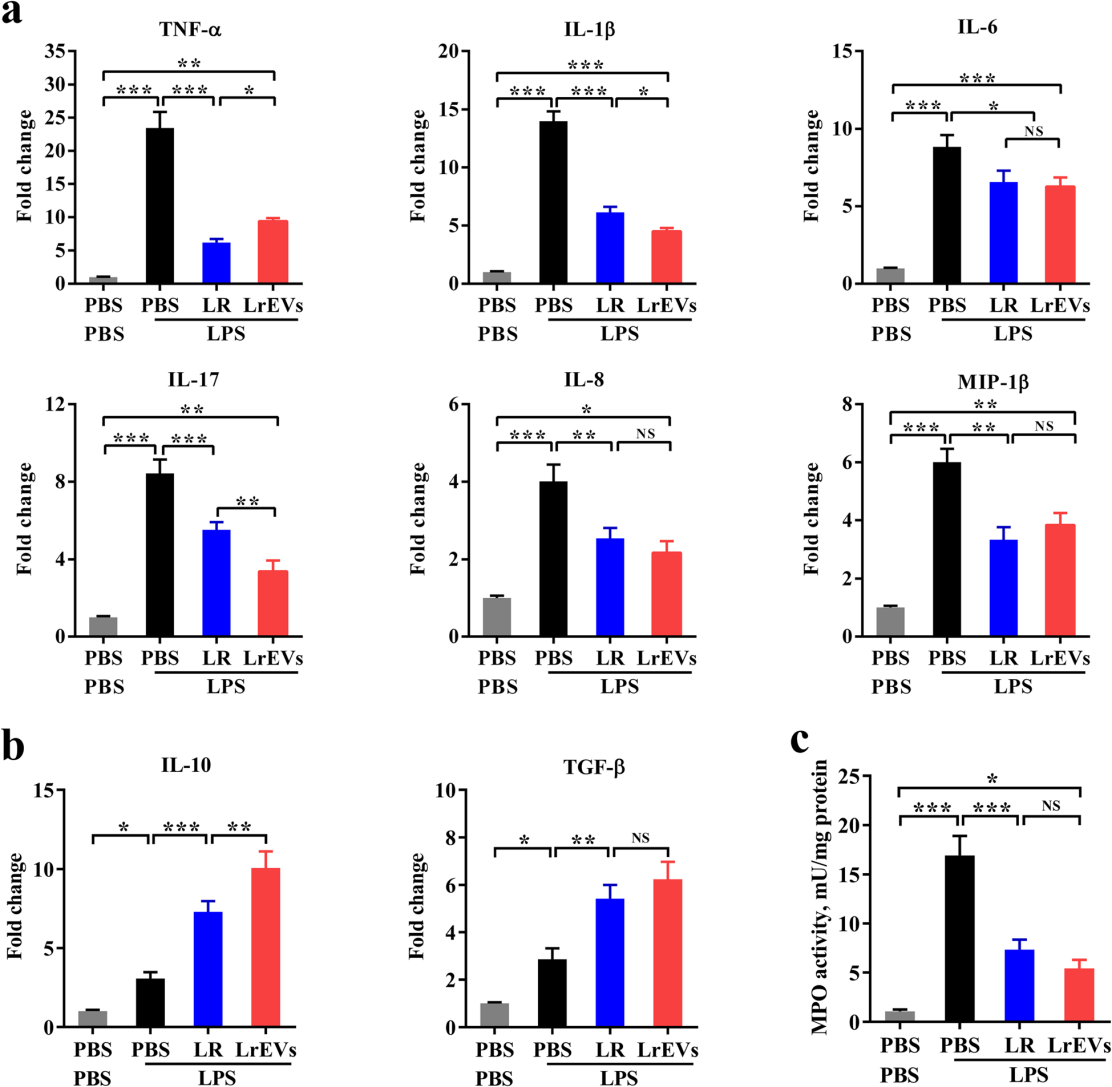
LrEVs modulate the gene expression of pro- and anti-inflammatory mediators in jejunum tissues. **a** LrEVs suppressed the LPS-induced gene expression of pro-inflammatory mediators, including pro-inflammatory cytokine genes *TNF-α*, *IL-1β*, *IL-6* and *IL-17*, and chemokine genes *IL-8* and *MIP-1β*. **b** LrEVs enhanced the expression of anti-inflammatory cytokine genes *IL-10* and *TGF-β* under the LPS-challenged condition. **c** LrEVs inhibited the LPS-induced activation of myeloperoxidase (MPO). Values are expressed as means ± SEM (*n* = 6). *P < 0.05; **P < 0.01; ***P < 0.001; NS, not significant

To investigate whether *L. reuteri* BBC3 and LrEVs have therapeutic effects, broiler chickens were given *L. reuteri* BBC3 or LrEVs by gavage during LPS-induced inflammation (Fig. 3a). Three days post the last LPS injection, ADWG (*P* < 0.001) and ADFI (*P* < 0.01) of LPS-only group (Fig. 3b; PBS + LPS) were significantly lower than those of PBS-only group (Fig. 3b; PBS + PBS); while F/G (*P* < 0.001) and the cumulative mortality (*P* < 0.01) of LPS-only group were significantly higher than those of PBS-only group. Remarkably, administration of both *L. reuteri* BBC3 and LrEVs significantly attenuated the reduced ADWG (*P* < 0.001) and ADFI (*P* < 0.01) and the increased F/G (*P* < 0.001) and mortality (*P* < 0.05) caused by LPS challenge (Fig. 3b; LR + LPS and LrEVs+LPS). According to histological analysis of jejunum tissues, obvious symptoms of intestinal injury, such as thinner mucosa, deformed crypt and many swelled and shed villus, were found in the LPS-only group; whereas, administration of *L. reuteri* BBC3 and LrEVs reduced the occurrence of intestinal injury (Fig. 3c). Additionally, LPS challenge significantly decreased the villus height (*P* < 0.001) and the value of VH/CD (*P* < 0.001), and significantly increased the crypt depth (*P* < 0.001); while administration of *L. reuteri* BBC3 and LrEVs significantly ameliorated the reduced villus height (*P* < 0.001) and VH/CD (*P* < 0.01) and the increased crypt depth (*P* < 0.01) (Fig. 4d). Interestingly, growth performance, mortality and indicators of villus morphology in the LrEVs+LPS group were not significantly different (*P* > 0.05) from those in the LR + PBS group, indicating that LrEVs had similar effects as *L. reuteri* BBC3 in attenuating LPS-induced intestinal injury.

### LrEVs modulate the gene expression of pro- and anti-inflammatory mediators in jejunum tissues

As shown in Fig. 4a**,** compared with the PBS-only group, the gene expression of several pro-inflammatory mediators, including *TNF-α* (*P* < 0.001), *IL-1β* (*P* < 0.001), *IL-6* (*P* < 0.001), *IL-8* (*P* < 0.001), *IL-17* (*P* < 0.001) and *MIP-1β* (*P* < 0.001), were significantly elicited in the LPS-only group. However, compared with the LPS-only group, administration of LrEVs significantly inhibited the gene expression of *TNF-α* (*P* < 0.001), *IL-1β* (*P* < 0.001), *IL-6* (*P* < 0.05), *IL-8* (*P* < 0.01), *IL-17* (*P* < 0.001) and *MIP-1β* (*P* < 0.01), implying that LrEVs might mediate protection in LPS-challenged chickens through suppressing these pro-inflammatory mediators. Besides, the gene expression of *IL-10* (*P* < 0.01) and *TGF-β* (*P* < 0.01) in the LrEVs+LPS group were significantly higher than those in the LPS-only group, indicating that these anti-inflammatory cytokines were also involved in the LrEVs-mediated regulation of intestinal inflammation. Meanwhile, LrEVs induced stronger suppression of *TNF-α* gene expression (*P* < 0.05) and enhancement of *IL-10* gene expression (*P <* 0.01) than LR. Furthermore, the MPO activity, representing neutrophil infiltration into inflamed tissues [35], was also significantly inhibited by *L. reuteri* BBC3 (*P <* 0.001) and LrEVs (*P <* 0.001). Accordingly, we conclude that the regulation of intestinal inflammation by *L. reuteri* BBC3 and LrEVs correlated with the suppression of pro-inflammatory cytokines and the activation of anti-inflammatory cytokines.

### LrEVs suppress NF-κB-dependent pro-inflammatory responses in LPS-activated chicken macrophages

**Fig. 5:**
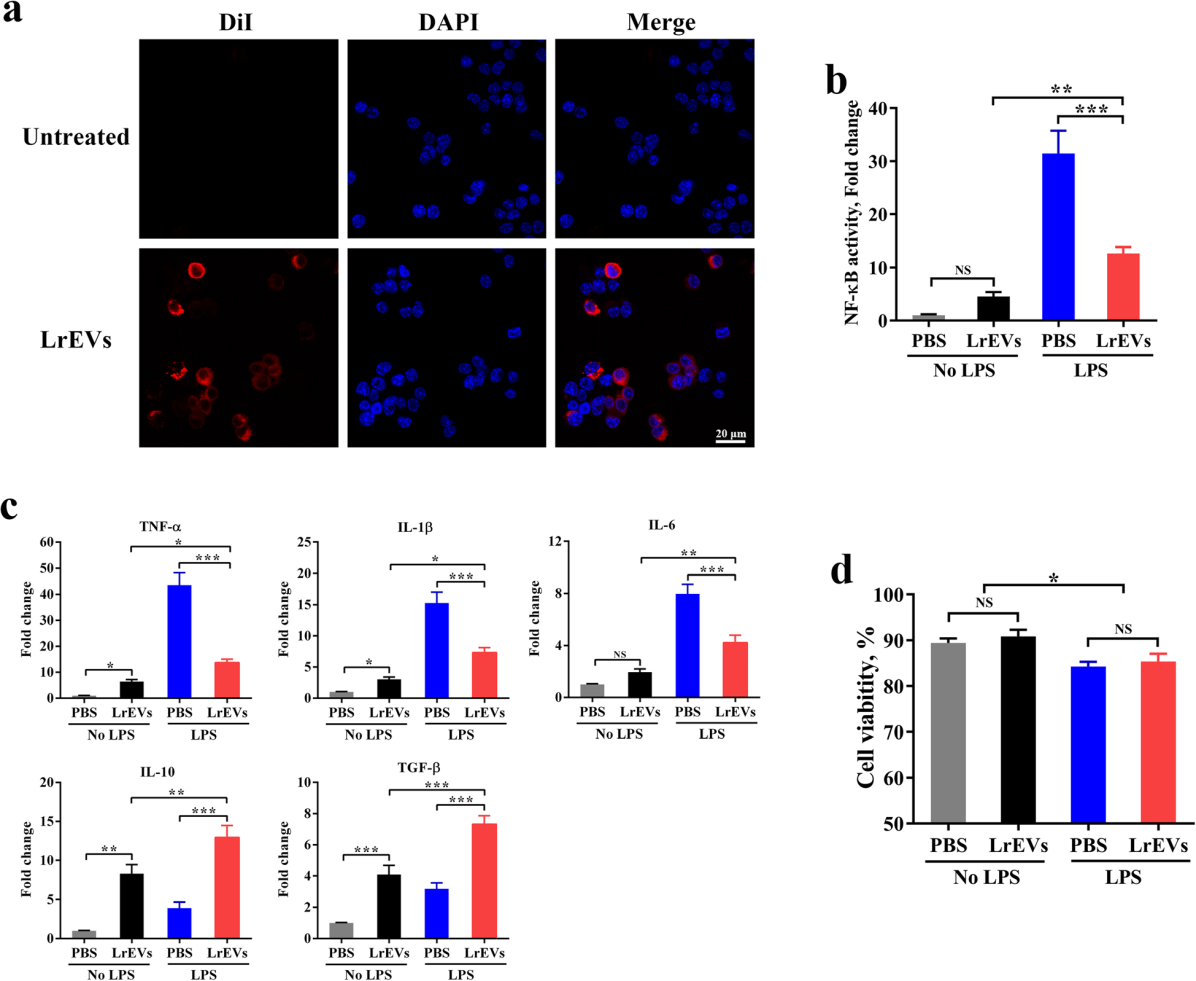
LrEVs suppress LPS-induced inflammatory responses in chicken HD11 macrophages (Mφ). **a** Confocal microscopy showed that LrEVs were internalized by HD11 cells. The HD11 cells were co-incubated with medium (row 1) and DiI-labeled LrEVs (row 2) for 6 h at 37 °C. LrEVs were labeled with DiI (red signal), and the cell nucleus was stained with DAPI (blue signal). **b** NF-κB p65 transcription factor activity in the cell nucleus from HD11 cells treated with the indicated conditions. HD11 cells (5 × 10^5^ cells/mL) were pretreated with PBS or LrEVs (10 μg/mL) for 12 h and stimulated with PBS or LPS (1 μg/mL) for 12 h. **c** The expression of pro-inflammatory cytokine genes *TNF-α*, *IL-1β*, *IL-6* and anti-inflammatory cytokine genes *IL-10* and *TGF-β* in HD11 cells treated with the indicated conditions. **d** Viability of HD11 cells determined by Trypan Blue dye exclusion assay. Data are representative of three independent experiments and expressed as means ± SEM. *P < 0.05; **P < 0.01; ***P < 0.001; NS, not significant

The similar protective effects mediated by *L. reuteri* BBC3 and LrEVs suggest that *L. reuteri* BBC3 might function via LrEVs on the host. To further investigate the roles of LrEVs in the microbiota-host interactions, we first examined whether LrEVs could be taken up by chicken HD11 Mφ *in vitro*. After co-incubation with HD11 cells for 6 h, the DiI-labeled LrEVs (red signals) were found in the cytoplasm of these cells, suggesting that LrEVs were internalized by Mφ (Fig. 5a). Moreover, pretreatment with LrEVs markedly inhibited the elevated activity of NF-κB (*P <* 0.001) in LPS-activated HD11 cells (Fig. 5b). Meanwhile, compared with stimulation with only PBS in HD11 cells, stimulation with only LPS significantly up-regulated the gene expression of NF-κB-dependent pro-inflammatory cytokines *TNF-α* (*P <* 0.001)*, IL-1β* (*P <* 0.001) and *IL-6* (*P <* 0.001); stimulation with only LrEVs significantly enhanced the gene expression of anti-inflammatory cytokines *IL-10* (*P <* 0.01) and *TGF-β* (*P <* 0.001). In LPS-challenged HD11 cells, pretreatment with LrEVs significantly induced lower gene expression of *TNF-α* (*P <* 0.001)*, IL-1β* (*P <* 0.001) and *IL-6* (*P <* 0.001) but higher gene expression of *IL-10* (*P <* 0.001) and *TGF-β* (*P <* 0.001) than pretreatment with PBS (Fig. 5c). Additionally, LrEVs did not affect (*P >* 0.05) the viability of PBS- or LPS-treated cells, thus ruling out the possibility that cytotoxicity was responsible for LrEVs-mediated suppression of NF-κB activity and pro-inflammatory cytokines. LPS stimulation, but not LrEVs-derived factors, was associated with the reduced cell viability (Fig. 5d). Collectively, these data demonstrate the capacity of internalized LrEVs in the suppression of NF-κB-dependent pro-inflammatory responses in LPS-activated Mφ.

### LrEVs induce anti-inflammatory responses in splenic lymphocytes through the activation of macrophages

**Fig. 6:**
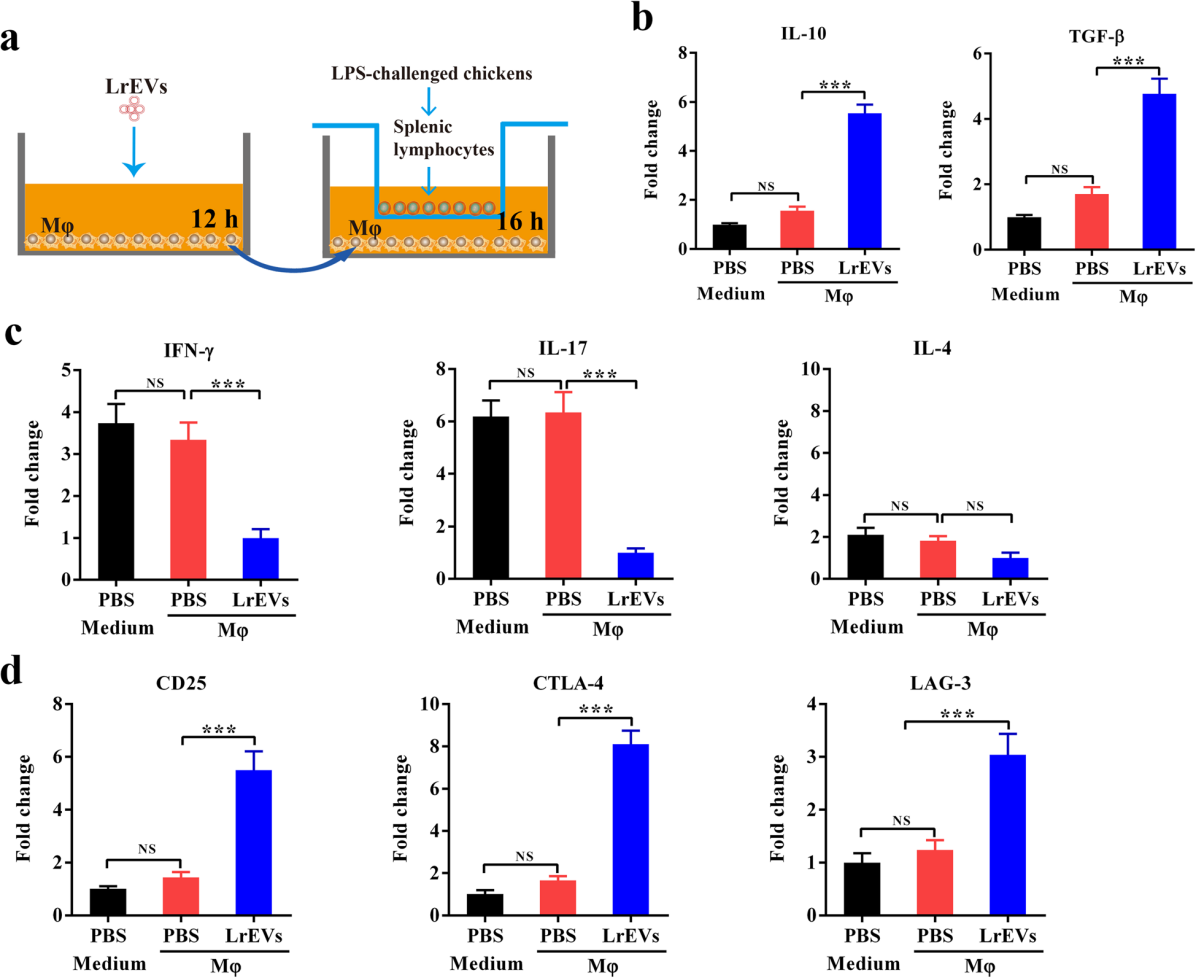
Treatment of macrophages with LrEVs induces anti-inflammatory responses in splenic lymphocytes. **a** Experimental schedule of Mφ-splenic lymphocytes coculture *in vitro* using a Transwell system. HD11 cells (5 × 10^5^ cells/mL) were pretreated with PBS or LrEVs (10 μg/mL) for 12 h, washed and added to the basolateral compartment of a 6-well plate. Splenic lymphocytes (5 × 10^5^ cells/mL) from LPS-challenged chickens were seeded on the apical compartment of 6-well Hanging Inserts and incubated with or without PBS- or LrEVs-pretreated HD11 cells for 12 h. **b** LrEVs increased the expression of anti-inflammatory cytokine genes *IL-10* and *TGF-β* in splenic lymphocytes of LPS-challenged chickens during *in vitro* coculture with HD11 cells. **c** LrEVs suppressed the expression of pro-inflammatory cytokine genes *IFN-γ* and *IL-17* in splenic lymphocytes of LPS-challenged chickens during *in vitro* coculture with HD11 cells. **d** Treatment of HD11 cells with LrEVs improved the gene expression of *CD25*, T-lymphocyte antigen 4 (*CTLA-4*) and lymphocyte activation gene 3 (*LAG-3*). Data are representative of three independent experiments and expressed as means ± SEM. ***P < 0.001; NS, not significant

To investigate the crosstalk between LrEVs, antigen-presenting cells (APCs) and adaptive immune cells, we performed an *in vitro* coculture assay of splenic lymphocytes with LrEVs-pretreated Mφ using a Transwell system (Fig. 6a). The HD11 cells were pretreated with LrEVs for 12 h, washed and subsequently cocultured with splenic lymphocytes of LPS-challenged chickens for 12 h. Splenic lymphocytes cocultured with LrEVs-pretreated Mφ showed the enhanced gene expression of *IL-10* (*P <* 0.001) and *TGF-β* (*P <* 0.001) compared to these cells cocultured with PBS-pretreated Mφ (Fig. 6b). Moreover, LrEVs significantly downregulated the gene expression of *IFN-γ* (*P <* 0.001) and *IL-17* (*P <* 0.001), the representative Th1 and Th17 cytokines, in splenic lymphocytes cocultured with Mφ; while the gene expression of IL-4 (*P >* 0.05), a representative Th2 cytokine, was not affected by LrEVs (Fig. 6c). Furthermore, LrEVs could also enhance the gene expression of *CD25* (*P <* 0.001), T-lymphocyte antigen 4 (*CTLA-4, P <* 0.001) and lymphocyte activation gene 3 (*LAG-3, P <* 0.001) in splenic lymphocytes cocultured with Mφ (Fig. 6d).

### Both vesicular proteins and nucleic acids are essential for the LrEVs-mediated immunoregulation

**Fig. 7:**
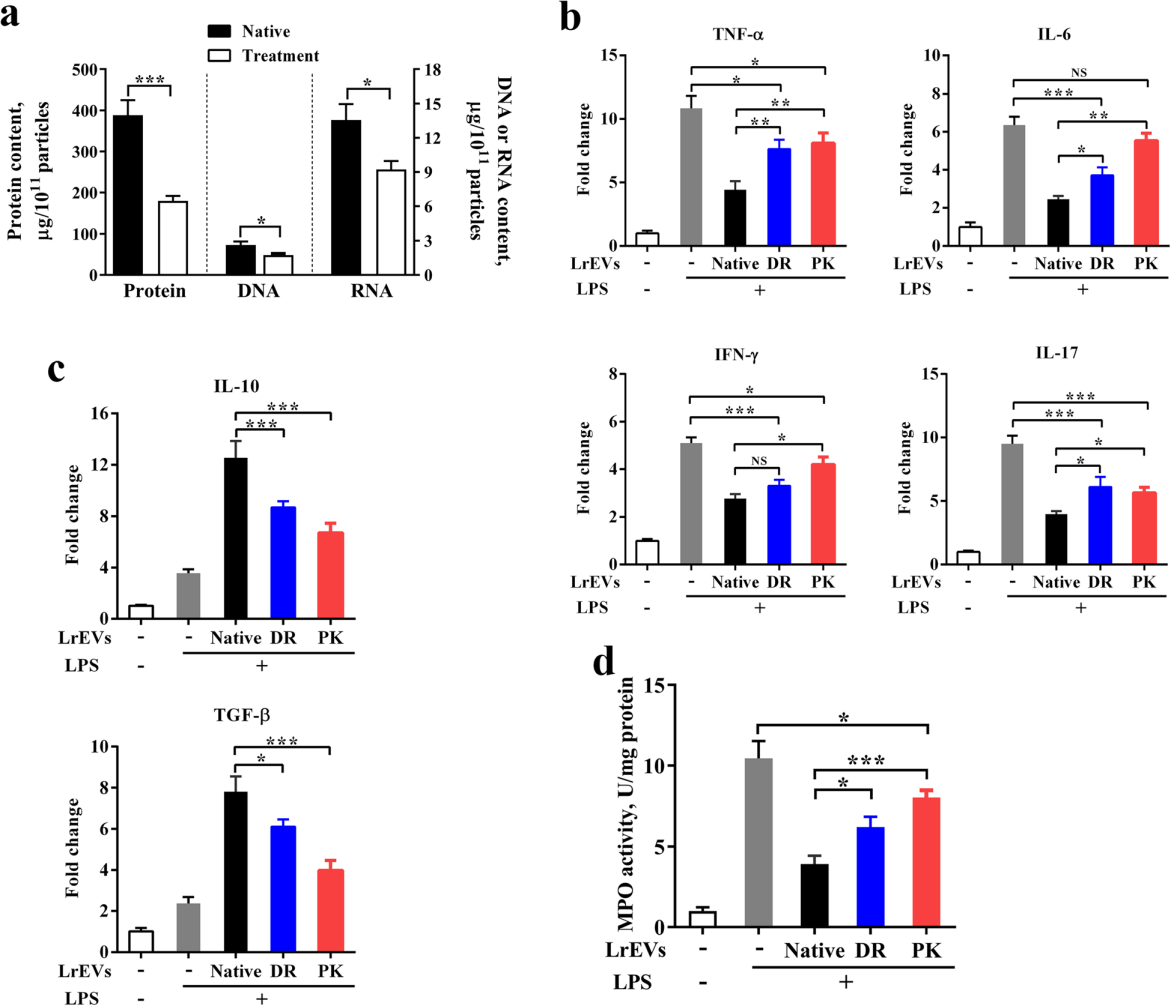
The reduced content of vesicular proteins and nucleic acids decreased the anti-inflammatory effects of LrEVs. **a** The content of proteins, DNA or RNA in the LrEVs treated with proteinase K, DNase I or RNase I (see the method). **b** The expression of pro-inflammatory cytokine genes *TNF-α*, *IL-6*, *IFN-γ* and *IL-17* in *ex vivo* jejunum explants. The explants were pretreated with native LrEVs (10 μg/mL), DNase I and RNase I-treated LrEVs (DR-LrEVs; 10 μg/mL) or proteinase K-agarose-treated LrEVs (PK-LrEVs; 10 μg/mL before proteinase K-agarose treatment) for 6 h and then stimulated with LPS (1 μg/mL) for 6 h. The gene expression of *IL-10* and *TGF-β* (**c**) and MPO activity (**d**) in *ex vivo* jejunum explants. Data are representative of three independent experiments and expressed as means ± SEM. *P < 0.05; **P < 0.01; ***P < 0.001; NS, not significant

We further used an *ex vivo* jejunum explant culture model to investigate the potential roles of these vesicular molecules in LrEVs-mediated regulation. LrEVs were treated with DNase I and RNase I (DR) and proteinase K-agarose (PK) to remove nucleic acids and proteins, respectively. The enzymes were inactivated at 75 °C for 1 h and proteinase K-agarose was additionally removed by centrifugation. Surprisingly, although these treatments significantly decreased the content of vesicular DNA (*P* < 0.05), RNA (*P* < 0.05) or proteins (*P* < 0.001) to some extent, they did not remove these components as much as expected, implying that the signals delivered by EVs are highly protected against exogenous proteases (Fig. 7a). Besides, the reduced content of nucleic acids or proteins might affect the functions of native LrEVs. Subsequently, a jejunum tissue culture system with LPS challenge was used as a closer model to the LPS-challenged condition *in vivo*. During *ex vivo* jejunum explant culture with LPS challenge, DR treatment significantly decreased the suppression of native LrEVs on the gene expression of *TNF-α* (*P* < 0.01), *IL-6* (*P* < 0.05) and *IL-17* (*P* < 0.05), and PK treatment significantly decreased the suppression of native LrEVs on the gene expression of *TNF-α* (*P* < 0.01), *IL-6* (*P* < 0.01), *IFN-γ* (*P* < 0.05) and *IL-17* (*P* < 0.05) (Fig. 7b). Additionally, DR- and PK-treated LrEVs showed a lower ability to induce the gene expression of *IL-10* (*P* < 0.001 and *P* < 0.001) and *TGF-β* (*P* < 0.05 and *P* < 0.001) compared to native LrEVs (Fig. 7c). Finally, DR and PK treatments also markedly reduced the ability of native LrEVs in the inhibition of MPO activity (*P* < 0.05 and *P* < 0.001), indicating that these treatments significantly decreased the suppressive activity of native LrEVs to inflammatory responses. Collectively, we conclude that both vesicular proteins and nucleic acids are crucial in the LrEVs-mediated immunoregulation.

## Discussion

Prohibition of antibiotic growth promoters has promoted the development of nutritional immunomodulators as a particularly attractive strategy to maintain gut health in today’s intensive poultry industry. Probiotics supplementation is becoming one of the most promising ways for the prevention and treatment of various inflammatory bowel diseases, due to its safety and ability in modulating intestinal immune homeostasis [36]. Previously, it has been illustrated that *L. reuteri* has the ability to regulate the host’s immune system in humans and animals [17, 18]. Consistent with these previous results, the present study demonstrated that administration of *L. reuteri* BBC3 attenuated the LPS-induced intestinal injury and inflammation in broiler chickens. We further revealed that EVs secreted by *L. reuteri* BBC3, a nanosized vesicle enriched with biological molecules of parental bacteria, could mediate bacteria-host interactions.

It is fundamentally important to understand the molecular mechanisms of the interaction of probiotics with the host to improve the application and clinical effectiveness of the probiotics. In addition to direct interaction with the host, the complex microbiota-host communication can also be mediated through active mediators secreted by microbiota, such as short-chain fatty acids, histamine and indole [37, 38]. Among various bacteria-derived mediators, EVs have been proved to play a key role in intercellular crosstalk or signaling [3, 39]. Bacterial EVs carry various bioactive molecules from parental bacteria, such as proteins, DNA, RNA and lipids. EVs are encapsulated vesicles with a nano-sized spherical lipid bilayer, thereby making them highly biocompatible and promoting the uptake by the host cells [4]. Much of the understanding of the role of bacterial EVs in mediating intercellular signaling is derived from numerous studies of pathogenic bacterial OMVs, showing that they are involved in the pathogenesis via delivering virulence factors to target cells [40, 41]. Only recently has it been increasingly interested in the mechanism of EVs secretion by which probiotics and commensal microbiota communicate with the host. Previous studies have revealed that EVs derived from several probiotics and commensal bacteria, such as *Bacteroides fragilis* [42], *Akkermansia muciniphila* [43], *Escherichia coli* Nissle 1917 [44], *Bifidobacterium longum* [45] and *Lactobacillus paracasei* [15], exerted similar effects as their parental bacteria. Consistent with these studies, we discovered that *L. reuteri* BBC3 could produce relatively high quantities of EVs, and treatment of broiler chicks with LrEVs reproduced the protective effects of *L. reuteri* BBC3 on LPS-induced lower performance and intestinal injury and inflammation.

Gram-negative bacterial LPS is known to induce acute inflammatory responses of the host by activating the NF-κB signaling pathway [46, 47]. The relatively high dose of LPS (500 μg/bird) was used to induce an *in vivo* model of intestinal inflammation in the present study. Previous studies have shown that the recovery of intestinal mucosal is relatively slow after a high dose of LPS challenge [48–50]. Consistent with these findings, our results showed that the high mortality and significant intestinal injury were observed in the LPS group continued until the third day after LPS injection. In the model of LPS-induced intestinal injury, many inflammatory mediators such as pro-inflammatory cytokines TNF-α, IL-1β and IL-6 and chemokines IL-8, were induced and released in the intestinal tissue [47, 51]. As a key mediator involved in inflammatory responses, IL-17 causes tissue damage via recruiting neutrophils into the gut and promoting the release of pro-inflammatory cytokines [42]. Macrophage inflammatory protein (MIP)-1β is particularly concerning chemokine associated with a variety of pro-inflammatory activities [52]. Our results showed that treatment of broiler chicks with LrEVs could attenuate LPS-induced intestinal inflammation by inhibiting the expression of these pro-inflammatory mediators and improving the expression of anti-inflammatory cytokines. Meanwhile, the different degrees of regulation of gene expression  indicated that the induced immunomodulatory effectiveness between bacterial EVs and the whole bacteria are not exactly equal. Despite this fact, these available results allow us to believe that EVs may act as an important mechanism of interaction between microbiota and the host.

Accordingly, it is conceivable that LrEVs could modulate the functions of the host’s immune cells. Indeed, we found that LrEVs could effectively be internalized by chicken Mφ and induce the gene expression of *IL-10* and *TGF-β* *in vitro*, suggesting that these nanovesicles directly interact with the innate immune cells. Several investigations have shown that EVs entered host cells via receptor-mediated pathways or lipid rafts [45, 53]. For Mφ, the uptake of EVs may also be through random phagocytosis, and the underlying mechanism requires to be further explored. Besides, it is well-known that Mφ is important participant in inflammatory responses, which produce multiple pro-inflammatory cytokines such as TNF-α, IL-1β and IL-6 [54]. After challenge with LPS, Mφ releases large quantities of these pro-inflammatory cytokines to promote inflammatory responses. In the model of LPS-activated Mφ used in this study, we observed that LrEVs could effectively suppress the gene expression of *TNF-α*, *IL-1β* and *IL-6*. Meanwhile, LrEVs also improved the gene expression of *IL-10* and *TGF-β*. IL-10 and TGF-β mainly mediate the negative regulation of inflammatory responses by suppressing the production of pro-inflammatory cytokines [55, 56]. Similar suppression of pro-inflammatory cytokines and activation of anti-inflammatory cytokines induced by *L. paracasei*-derived EVs have also been observed with LPS-challenged human colon cancer cells [15]. Furthermore, several studies have demonstrated that probiotics-derived EVs controlled inflammatory responses by modulating the cytokine production in the host’s immune cells [31, 42]. Collectively, these findings support the suggestion that LrEVs can directly control the increased immune responses in inflammatory cells by modulating cytokine gene expression.

Previous studies have shown that *L. reuteri* has the ability to induce the development of effector T cells and forkhead box P3^+^ (Foxp3^+^) Tregs in the mammalian gut [21, 22]. Shen et al. revealed that gut commensal *B. fragilis*-derived EVs could induce the production of IL-10 in mouse DCs, which in turn promotes the development of Tregs [42]. Similarly, we found that LrEVs could elicit *IL-10* gene expression in the cultured chicken Mφ alone, and the gene expression of *IL-10* and *TGF-β* in splenic lymphocytes were also up-regulated when they were cocultured with LrEVs-stimulated Mφ. These findings indicate that LrEVs can induce anti-inflammatory responses in splenic lymphocytes through the activation of Mφ. Moreover, IFN-γ and IL-17 are representative Th1 and Th17 cytokines, respectively, which are mainly involved in the inflammatory responses [57]. Th1 and Th17-mediated inflammatory responses can be dampened by the Treg-derived IL-10 [57, 58]. The gene expression of *IFN-γ* and *IL-17* in splenic lymphocytes were down-regulated in this study under the cocultured condition with LrEVs-pretreated Mφ, implying that LrEVs can also inhibit the pro-inflammatory responses in splenic lymphocytes through the activation of Mφ. Furthermore, Foxp3^+^ Tregs play a curial role in inhibiting inflammatory responses in the intestine by secreting the anti-inflammatory cytokines IL-10 and TGF-β [59]. Mammalian Foxp3^+^ Tregs can exert the immunosuppressive effect on effective T cells by increasing the gene expression of *CTLA-4* [60]. The presence of *LAG-3* in mammalian Tregs can inhibit the maturation of DCs, thus inducing the Treg-mediated suppression [61]. According to the results of splenic lymphocyte sub-population analysis with flow cytometer by Shanmugasundaram et al., the gene expression of *CTLA-4* and *LAG-3* can be served as markers for the development of chicken CD4^+^CD25^+^ cells [62]. Although no Foxp3 ortholog genes have been identified in chickens to date, CD4^+^CD25^+^ cells from chicken spleen tissue have been shown to have the immunosuppressive and cytokine-producing properties of mammalian Foxp3^+^Tregs [62]. The gene expression of *CD25*, *CTLA-4* and *LAG-3* in splenic lymphocytes were enhanced in this study after coculture with LrEVs-pretreated Mφ, implying that CD4^+^CD25^+^ cells may participate in the LrEVs-mediated immunomodulation. However, whether *IL-10* activated by LrEVs in Mφ is involved in the immunomodulatory activities of LrEVs-stimulated Mφ and exactly which lymphocyte subtypes are involved in the LrEVs-mediated immunomodulation remains to be further studied.

Although the LrEVs-mediated immunomodulation was verified in this study, it is challenging to determine which specific molecules play the most important role. Among the compositions of bacterial EVs, proteins account for the largest proportion and mediate many functions of EVs [1]. Previously, it has been illustrated that certain proteins isolated from the probiotic-derived EVs exert a similar effect of the intact EVs. *B. longum* KACC 91563-derived EVs contain a protein ESBP that can induce the beneficial effect of the bacterial EVs [45]. EVs derived from *L. casei* BL23 carry several proteins associated with the probiotic effect of the bacteria, such as p40 and p75 [12]. In the present study, we characterized several proteins that are potentially associated with immune regulation in LrEVs, such as glucosyltransferase, serine protease, 60 kDa chaperonin, elongation factor Tu and inositol polyphosphate phosphatase 1. Bacterial glucosyltransferase may glycosylate the host target proteins, thus providing glycan decorations that can activate the DC-SIGN signaling in DCs and induce the production of IL-10 [63, 64]. Serine protease secreted by *L.* paracase has shown to be able to inhibit inflammatory responses by selectively targeting pro-inflammatory chemokines [65]. Bacterial 60 kDa chaperonin, an heat shock protein analogue, may induce the development of CD4^+^CD25^+^ Tregs and mediate TLR-2-dependent immunoregulation [66, 67]. Elongation factor Tu and inositol polyphosphate phosphatase 1 have also been observed to be involved in the intercellular signaling [68, 69]. Additionally, nucleic acid molecules derived from probiotics have also been proven to exert suppressive activity against inflammatory responses. Probiotics- and commensal bacteria-derived CpG DNA can mediate anti-inflammatory responses via Toll-like receptor 9 signaling [36, 70]. *L. gasseri*-derived RNA can inhibit inflammatory responses through a MyD88-dependent signaling pathway [71]. The study presented here confirmed that proteins and nucleic acids present in the LrEVs are essential for the LrEVs-mediated immunomodulation. Further studies will aim to explore the exact molecules involved in the immunoregulatory effect of LrEVs.

**Fig. 8:**
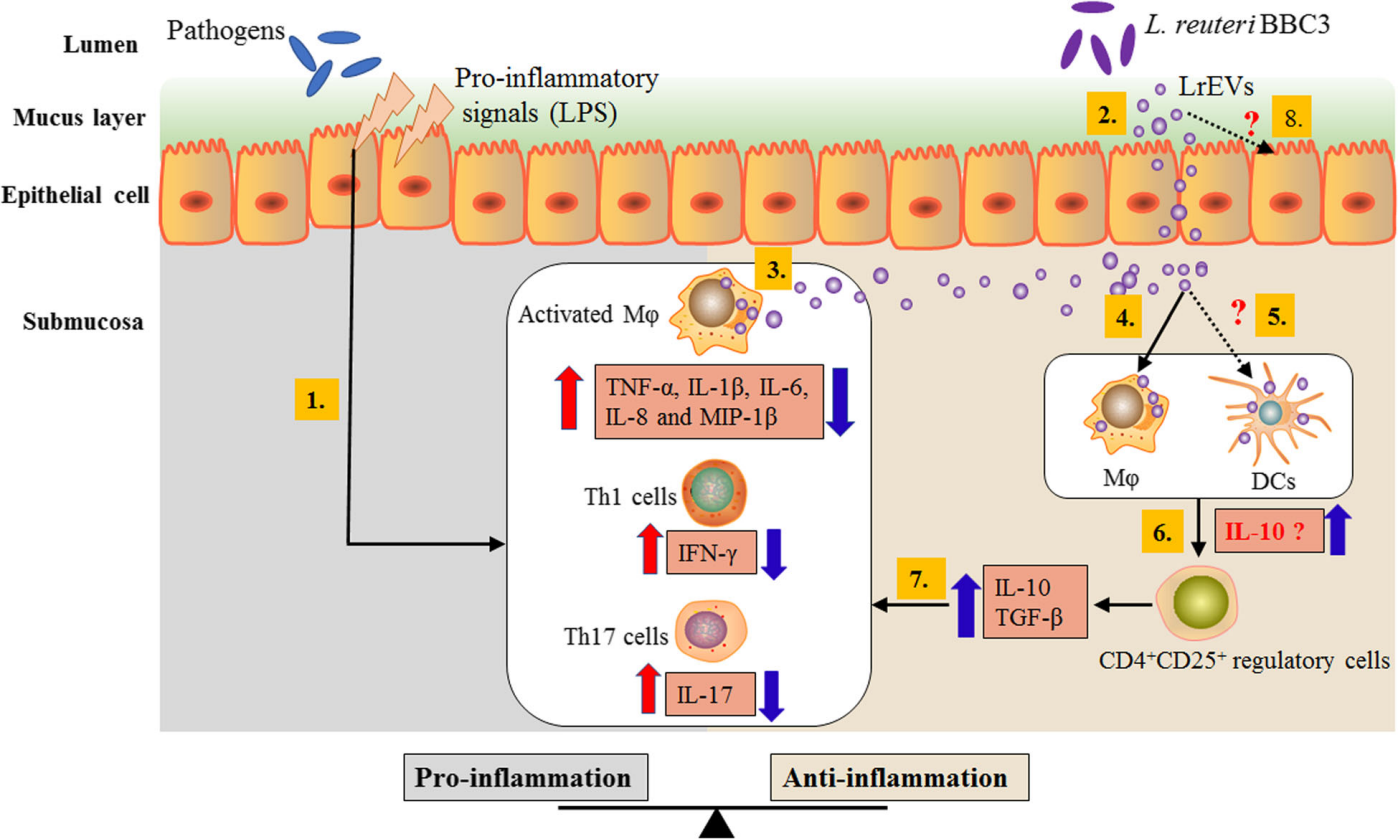
Proposed mechanism of LrEVs-mediated bacteria-host crosstalk to drive the intestinal immune homeostasis against pathogens-induced inflammation in the chicken model. In inflammatory bowel disease, pathogens remarkably proliferate in the gut lumen and produce pro-inflammatory signals, such as lipopolysaccharide (LPS), which activate inflammatory cells, including macrophages (Mφ), Th1 and Th17 cells, to produce pro-inflammatory responses (1) [57]. *L. reuteri* BBC3 releases nanosized and highly biocompatible EVs that can drive the long-distance transport of interior molecules throughout the intracellular compartments in a concentrated, protected and targeted manner (2) [72]. These vesicles can suppress the pro-inflammatory mediators produced by inflammatory cells (activated Mφ) (3), and activate innate immune cells, including naïve Mφ (4) and possibly dendritic cells (DCs) (5), to produce immunoregulatory cytokines (possibly including IL-10) (6) that induce the development of immunoregulatory CD4^+^CD25^+^ cells. These resulting CD4^+^CD25^+^ cells can produce anti-inflammatory cytokines IL-10 and TGF-β that inhibit the production of pro-inflammatory cytokines (7) [62]. Further studies are required to investigate the potential interactions between LrEVs and other intestinal immune cells, especially DCs (5) and epithelial cells (8)

Due to the unique nano-scale structure of the lipid membrane-encapsulated vesicles, EVs can drive the long-distance transport of interior molecules throughout the intracellular compartments in a concentrated, protected and targeted manner [72]. According to the available results, we present a possible mechanism of LrEVs-mediated intestinal immune homeostasis against LPS-induced inflammation in the gut of the chicken model (Fig. 8). LrEVs are transported through intestinal epithelial cells, then suppress inflammatory responses in the activated Mφ and effector T cells, and promote the development of immunoregulatory CD4^+^CD25^+^ cells and the induction of anti-inflammatory cytokines. Further studies are needed to investigate the potential interactions between the LrEVs and other intestinal immune cells, especially epithelial cells and DCs.

## Conclusion

In summary, we revealed that treatment of broiler chicks with LrEVs recapitulated the suppression of *L. reuteri* BBC3 on the LPS-induced intestinal injury and inflammation by modulating the gene expression of cytokines. We confirmed that LrEVs could mediate immune responses in LPS-activated Mφ and splenic lymphocytes cocultured with Mφ *in vitro*. Moreover, we demonstrated that vesicular proteins and nucleic acids were required for LrEVs-mediated bacteria-host interactions. To the best of our knowledge, this study is the first to reveal that the EVs secreted by *L. reuteri* played an important role in immune regulation and disease protection in the gut of the chicken model.

## Supplementary Information

**Additional file 1: Table S1.** Primers used for quantitative real-time PCR in this study.

**Additional file 2: Table S2.** A total of 92 overlapping proteins identified in triplicate biological samples of LrEVs.

## Data Availability

All data generated or analyzed during this study are included in this published article and its additional file.

## Abbreviations

MVs: Membrane vesicles
EVs: Extracellular vesicles
OMVs: Outer membrane vesicles
TNF-α: Tumour necrosis factor α
IL: Interleukin
DCs: Dendritic cells
LPS: Lipopolysaccharide
Mφ: Macrophages
Tregs: Regulatory T cells
LrEVs: *L. reuteri*-derived EVs
PBS: Phosphate buffer saline
NTA: Nanoparticle tracking analysis
LC-MS/MS: Tandem mass spectrometry coupled online to the UPLC
GO: Gene Ontology
ADWG: Average daily weight gain
ADFI: Average daily feed intake
F/G: Feed gain ratio
DiI: Dialkylcarbocyanine iodide
DAPI: 4′, 6-Diamidino-2-phenylindole
FBS: Fetal bovine serum
MPO: Myeloperoxidase
qRT-PCR: Quantitative real-time PCR
SEM: Standard error of the mean
MIP: Macrophage inflammatory proteins
APCs: Antigen-presenting cells
Th: T helper
Foxp3: Forkhead box P3
CTLA-4: T-lymphocyte antigen 4
LAG-3: Lymphocyte activation gene 3

## References

[1] Brown L, Wolf JM, Prados-Rosales R, Casadevall A. Through the wall: extracellular vesicles in gram-positive bacteria, mycobacteria and fungi. Nat Rev Microbiol. 2015;13(10):620–30. 10.1038/nrmicro3480 PMC4860279 26324094

[2] Yáñez-Mó M, Siljander PR-M, Andreu Z, Zavec AB, Borràs FE, Buzas EI (2015). Biological properties of extracellular vesicles and their physiological functions. J Extracell Vesicles.

[3] Kim JH, Lee J, Park J, Gho YS (2015). Gram-negative and gram-positive bacterial extracellular vesicles. Semin Cell Dev Biol.

[4] Schwechheimer C, Kuehn MJ. Outer-membrane vesicles from gram-negative bacteria: biogenesis and functions. Nat Rev Microbiol. 2015;13(10):605–19. 10.1038/nrmicro3525 PMC5308417 26373371

[5] Acevedo R, Fernandez S, Zayas C, Acosta A, Elena Sarmiento M, Ferro VA (2014). Bacterial outer membrane vesicles and vaccine applications. Front Immunol.

[6] van der Pol L, Stork M, van der Ley P (2015). Outer membrane vesicles as platform vaccine technology. Biotechnol J.

[7] Lee EY, Choi DY, Kim DK, Kim JW, Park JO, Kim S (2009). Gram-positive bacteria produce membrane vesicles: proteomics-based characterization of *Staphylococcus aureus*-derived membrane vesicles. Proteomics..

[8] Rivera J, Cordero RJB, Nakouzi AS, Frases S, Nicola A, Casadevall A (2010). *Bacillus anthracis* produces membrane-derived vesicles containing biologically active toxins. P Natl Acad Sci USA..

[9] Olaya-Abril A, Prados-Rosales R, McConnell MJ, Martin-Pena R, Gonzalez-Reyes JA, Jimenez-Munguia I (2014). Characterization of protective extracellular membrane-derived vesicles produced by *Streptococcus pneumoniae*. J Proteome.

[10] Brown L, Kessler A, Cabezas-Sanchez P, Luque-Garcia JL, Casadevall A (2014). Extracellular vesicles produced by the gram-positive bacterium *Bacillus subtilis* are disrupted by the lipopeptide surfactin. Mol Microbiol.

[11] Jiang Y, Kong Q, Roland KL, Curtiss R (2014). Membrane vesicles of *Clostridium perfringens* type a strains induce innate and adaptive immunity. Int J Med Microbiol.

[12] Rubio APD, Martinez JH, Casillas DCM, Leskow FC, Piuri M, Perez OE (2017). *Lactobacillus casei* BL23 produces microvesicles carrying proteins that have been associated with its probiotic effect. Front Microbiol.

[13] Li M, Lee K, Hsu M, Nau G, Mylonakis E, Ramratnam B (2017). *Lactobacillus*-derived extracellular vesicles enhance host immune responses against vancomycin-resistant enterococci. BMC Microbiol.

[14] Dean SN, Leary DH, Sullivan CJ, Oh E, Walper SA (2019). Isolation and characterization of *Lactobacillus*-derived membrane vesicles. Sci Rep-Uk..

[15] Choi JH, Moon CM, Shin T-S, Kim EK, McDowell A, Jo M-K (2020). *Lactobacillus paracasei*-derived extracellular vesicles attenuate the intestinal inflammatory response by augmenting the endoplasmic reticulum stress pathway. Exp Mol Med.

[16] Grande R, Celia C, Mincione G, Stringaro A, Di Marzio L, Colone M (2017). Detection and physicochemical characterization of membrane vesicles (MVs) of *Lactobacillus reuteri* DSM 17938. Front Microbiol.

[17] Mu Q, Tavella VJ, Luo XM (2018). Role of *Lactobacillus reuteri* in human health and diseases. Front Microbiol.

[18] Walter J, Britton RA, Roos S (2011). Host-microbial symbiosis in the vertebrate gastrointestinal tract and the *Lactobacillus reuteri* paradigm. P Natl Acad Sci USA.

[19] Christensen HR, Frøkiær H, Pestka JJ (2002). *Lactobacilli* differentially modulate expression of cytokines and maturation surface markers in murine dendritic cells. J Immunol.

[20] Lin YP, Thibodeaux CH, Peña JA, Ferry GD, Versalovic J (2008). Probiotic *Lactobacillus reuteri* suppress proinflammatory cytokines via c-Jun. Inflamm Bowel Dis.

[21] Liu YY, Tran DQ, Fatheree NY, Rhoads JM (2014). *Lactobacillus reuteri* DSM 17938 differentially modulates effector memory T cells and Foxp3^+^ regulatory T cells in a mouse model of necrotizing enterocolitis. Am J Physiol-Gastr L.

[22] He B, Hoang TK, Wang T, Ferris M, Taylor CM, Tian X, et al. Resetting microbiota by *Lactobacillus reuteri* inhibits T reg deficiency–induced autoimmunity via adenosine A_2A_ receptors. J Exp Med. 2016;214(1):107–23. 10.1084/jem.20160961 PMC5206500 27994068

[23] Hilmi HTA, Surakka A, Apajalahti J, Saris PE (2007). Identification of the most abundant *Lactobacillus* species in the crop of 1- and 5-week-old broiler chickens. Appl Environ Microbiol.

[24] Mappley LJ, Tchórzewska MA, Nunez A, Woodward MJ, Bramley PM, La Ragione RM (2013). Oral treatment of chickens with *Lactobacillus reuteri* LM1 reduces *Brachyspira pilosicoli*-induced pathology. J Med Microbiol.

[25] Nakphaichit M, Sobanbua S, Siemuang S, Vongsangnak W, Nakayama J, Nitisinprasert S (2019). Protective effect of *Lactobacillus reuteri* KUB-AC5 against *Salmonella Enteritidis* challenge in chickens. Benef Microbes.

[26] Prados-Rosales R, Brown L, Casadevall A, Montalvo-Quirós S, Luque-Garcia JL (2014). Isolation and identification of membrane vesicle-associated proteins in gram-positive bacteria and mycobacteria. MethodsX..

[27] Hu R, Li J, Zhao Y, Lin H, Liang L, Wang M, et al. Exploiting bacterial outer membrane vesicles as a cross-protective vaccine candidate against avian pathogenic *Escherichia coli* (APEC). Microb Cell Fact. 2020;19(1):119. 10.1186/s12934-020-01372-7 PMC7268718 32493405

[28] Lee WH, Choi HI, Hong SW, Kim KS, Gho YS, Jeon SG (2015). Vaccination with *Klebsiella pneumoniae*-derived extracellular vesicles protects against bacteria-induced lethality via both humoral and cellular immunity. Exp Mol Med.

[29] Chen Y, Zhang H, Cheng Y, Li Y, Wen C, Zhou Y (2018). Dietary L-threonine supplementation attenuates lipopolysaccharide-induced inflammatory responses and intestinal barrier damage of broiler chickens at an early age. Brit J Nutr.

[30] Beug H, von Kirchbach A, Döderlein G, Conscience J-F, Graf T (1979). Chicken hematopoietic cells transformed by seven strains of defective avian leukemia viruses display three distinct phenotypes of differentiation. Cell..

[31] Forsberg MM, Björkander S, Pang Y, Lundqvist L, Ndi M, Ott M (2019). Extracellular membrane vesicles from *Lactobacilli* dampen IFN-γ responses in a monocyte-dependent manner. Sci Rep-Uk.

[32] Zhang Q, Chen X, Eicher S, Ajuwon K, Applegate T (2017). Effect of threonine on secretory immune system using a chicken intestinal *ex vivo* model with lipopolysaccharide challenge. Poultry Sci..

[33] Livak KJ, Schmittgen TD (2001). Analysis of relative gene expression data using real-time quantitative PCR and the 2^−ΔΔCt^ method. Methods..

[34] Strober W (1997). Trypan blue exclusion test of cell viability. Current Protocols in Immunology.

[35] Jiang LL, Shen YY, Guo DF, Yang DY, Liu JJ, Fei XF (2016). EpCAM-dependent extracellular vesicles from intestinal epithelial cells maintain intestinal tract immune balance. Nat Commun.

[36] Walker WA (2008). Mechanisms of action of probiotics. Clin Infect Dis.

[37] Thaiss CA, Zmora N, Levy M, Elinav E (2016). The microbiome and innate immunity. Nature..

[38] Macia L, Nanan R, Hosseini-Beheshti E, Grau GE (2020). Host- and microbiota-derived extracellular vesicles, immune function, and disease development. Int J Mol Sci.

[39] Kaparakis-Liaskos M, Ferrero RL (2015). Immune modulation by bacterial outer membrane vesicles. Nat Rev Immunol..

[40] Kesty NC, Mason KM, Reedy M, Miller SE, Kuehn MJ (2004). Enterotoxigenic *Escherichia coli* vesicles target toxin delivery into mammalian cells. EMBO J.

[41] Rueter C, Bielaszewska M (2020). Secretion and delivery of intestinal pathogenic *Escherichia coli* virulence factors via outer membrane vesicles. Front Cell Infect Mi.

[42] Shen Y, Torchia MLG, Lawson GW, Karp CL, Ashwell JD, Mazmanian SK (2012). Outer membrane vesicles of a human commensal mediate immune regulation and disease protection. Cell Host Microbe.

[43] Kang CS, Ban M, Choi EJ, Moon HG, Jeon JS, Kim DK (2013). Extracellular vesicles derived from gut microbiota, especially *Akkermansia muciniphila*, protect the progression of dextran sulfate sodium-induced colitis. PLoS One.

[44] Fábrega MJ, Aguilera L, Giménez R, Varela E, Cañas MA, Antolin M (2016). Activation of immune and defense responses in the intestinal mucosa by outer membrane vesicles of commensal and probiotic *Escherichia coli* strains. Front Microbiol.

[45] Kim J-H, Jeun E-J, Hong C-P, Kim S-H, Jang MS, Lee E-J, et al. Extracellular vesicle–derived protein from *Bifidobacterium longum* alleviates food allergy through mast cell suppression, J Allergy Clin Immun. 2016;137(2):507–16 e8. 10.1016/j.jaci.2015.08.016 26433560

[46] Guijarro-Muñoz I, Compte M, Álvarez-Cienfuegos A, Álvarez-Vallina L, Sanz L (2014). Lipopolysaccharide activates toll-like receptor 4 (TLR4)-mediated NF-κB signaling pathway and proinflammatory response in human pericytes. J Biol Chem.

[47] Liu S, Song M, Yun W, Lee J, Kim H, Cho J (2019). Effect of carvacrol essential oils on immune response and inflammation-related genes expression in broilers challenged by lipopolysaccharide. Poultry Sci.

[48] Hu X, Guo Y, Li J, Yan G, Bun S, Huang B (2011). Effects of an early lipopolysaccharide challenge on growth and small intestinal structure and function of broiler chickens. Can J Anim Sci.

[49] Li R, Song Z, Zhao J, Huo D, Fan Z, Hou D-X (2018). Dietary L-theanine alleviated lipopolysaccharide-induced immunological stress in yellow-feathered broilers. Anim Nutr.

[50] Yang L, Liu G, Zhu X, Luo Y, Shang Y, Gu X-L (2019). The anti-inflammatory and antioxidant effects of leonurine hydrochloride after lipopolysaccharide challenge in broiler chicks. Poultry Sci..

[51] Li R, Li J, Zhang S, Mi Y, Zhang C (2018). Attenuating effect of melatonin on lipopolysaccharide-induced chicken small intestine inflammation. Poultry Sci..

[52] Abe M, Hiura K, Wilde J, Moriyama K, Hashimoto T, Ozaki S (2002). Role for macrophage inflammatory protein (MIP)-1α and MIP-1β in the development of osteolytic lesions in multiple myeloma. Blood..

[53] Kaparakis M, Turnbull L, Carneiro L, Firth S, Coleman HA, Parkington HC (2010). Bacterial membrane vesicles deliver peptidoglycan to NOD1 in epithelial cells. Cell Microbiol.

[54] Murray PJ, Wynn TA (2011). Protective and pathogenic functions of macrophage subsets. Nat Rev Immunol.

[55] Letterio JJ, Roberts AB (1998). Regulation of immune responses by TGF-β. Annu Rev Immunol.

[56] Fiorentino DF, Zlotnik A, Mosmann T, Howard M, O'garra A (1991). IL-10 inhibits cytokine production by activated macrophages. J Immunol.

[57] Arrieta M-C, Finlay BB (2012). The commensal microbiota drives immune homeostasis. Front Immunol.

[58] Weaver CT, Harrington LE, Mangan PR, Gavrieli M, Murphy KM (2006). Th17: an effector CD4 T cell lineage with regulatory T cell ties. Immunity..

[59] Vignali DAA, Collison LW, Workman CJ (2008). How regulatory T cells work. Nat Rev Immunol..

[60] Wing K, Onishi Y, Prieto-Martin P, Yamaguchi T, Miyara M, Fehervari Z (2008). CTLA-4 control over Foxp3^+^ regulatory T cell function. Science..

[61] Huang C-T, Workman CJ, Flies D, Pan X, Marson AL, Zhou G (2004). Role of LAG-3 in regulatory T cells. Immunity..

[62] Shanmugasundaram R, Selvaraj RK (2011). Regulatory T cell properties of chicken CD4^+^CD25^+^ cells. J Immunol.

[63] van Baarlen P, Wells JM, Kleerebezem M (2013). Regulation of intestinal homeostasis and immunity with probiotic *Lactobacilli*. Trends Immunol.

[64] Gao L, Song QT, Liang H, Zhu YT, Wei TT, Dong N, et al. *Legionella* effector SetA as a general O-glucosyltransferase for eukaryotic proteins. Nat Chem Biol. 2019;15(3):213–6. 10.1038/s41589-018-0189-y 30617292

[65] von Schillde MA, Hormannsperger G, Weiher M, Alpert CA, Hahne H, Bauerl C (2012). Lactocepin secreted by *Lactobacillus* exerts anti-inflammatory effects by selectively degrading proinflammatory chemokines. Cell Host Microbe.

[66] Wang X, Zhou S, Chi Y, Wen X, Hoellwarth J, He L (2009). CD4^+^CD25^+^ Treg induction by an HSP60-derived peptide SJMHE1 from *Schistosoma japonicum* is TLR2 dependent. Eur J Immunol.

[67] Al-Nedawi K, Mian MF, Hossain N, Karimi K, Mao YK, Forsythe P (2015). Gut commensal microvesicles reproduce parent bacterial signals to host immune and enteric nervous systems. FASEB J.

[68] Granato D, Bergonzelli GE, Pridmore RD, Marvin L, Rouvet M, Corthesy-Theulaz IE. Cell surface-associated elongation factor Tu mediates the attachment of *Lactobacillus johnsonii* NCC533 (La1) to human intestinal cells and mucins. Infect Immun. 2004;72(4):2160–9. 10.1128/IAI.72.4.2160-2169.2004 PMC375183 15039339

[69] Stentz R, Osborne S, Horn N, Li AWH, Hautefort I, Bongaerts R (2014). A bacterial homolog of a eukaryotic inositol phosphate signaling enzyme mediates cross-kingdom dialog in the mammalian gut. Cell Rep.

[70] Rachmilewitz D, Katakura K, Karmeli F, Hayashi T, Reinus C, Rudensky B (2004). Toll-like receptor 9 signaling mediates the anti-inflammatory effects of probiotics in murine experimental colitis. Gastroenterology..

[71] Yoshida A, Yamada K, Yamazaki Y, Sashihara T, Ikegami S, Shimizu M, et al. *Lactobacillus**gasseri* OLL2809 and its RNA suppress proliferation of CD4^+^ T cells through a MyD88-dependent signaling pathway. Immunology. 2011;133(4):442–51. 10.1111/j.1365-2567.2011.03455.x PMC3143356 21627651

[72] Kulp A, Kuehn MJ (2010). Biological functions and biogenesis of secreted bacterial outer membrane vesicles. Annu Rev Microbiol.

