# Supplementary material for: Lactobacillus reuteri-derived extracellular vesicles maintain intestinal immune homeostasis against lipopolysaccharide-induced inflammatory responses in broilers
Source: J Anim Sci Biotechnol. 2021 Feb 17;12:25. doi: 10.1186/s40104-020-00532-4 (PMC7888134; doi:10.1186/s40104-020-00532-4)
Supplement: Supplementary file 1 — Additional file 1: Table S1. Primers used for quantitative real-time PCR in this study. [file 40104_2020_532_MOESM1_ESM.docx]

**Table S1. Primers used for quantitative real-time PCR in this study. ^a^**

| **Gene** | **Acc. No.** | **Primers sequence (5’→3’)** | **Reference** |
| --- | --- | --- | --- |
| *β-actin* | NM_205518 | F: ACACCCACACCCCTGTGATGAA | Li et al. [1] |
|  |  | R: TGCTGCTGACACCTTCACCATTC |  |
| *TNF-α* | JN942589.1 | F: AGTGCTGTTCTATGACCGCC | Li et al. [1] |
|  |  | R: CGCTCCTGACTCATAGCAGA |  |
| *IL-1β* | NM_204524.1 | F: ATGACCAAACTGCTGCGGAG | Bolha et al.[2] |
|  |  | R: GTCGCTGTCAGCAAAGTCCC |  |
| *IL-4* | NM_001007079.1 | F: TCTTCCTCAACATGCGTCAG | Li et al. [1] |
|  |  | R: TGGTGGAAGAAGGTACGTAGG |  |
| *IL-6* | NM_204628.1 | F: CTCCTCGCCAATCTGAAGTC | Li et al. [1] |
|  |  | R: GGCACTGAAACTCCTGGTCT |  |
| *IL-8* | NM_205498.1 | F: CTGCGGTGCCAGTGCATTAG | Liu et al. [3] |
|  |  | R: GCACACCTCTCTTCCATCC |  |
| *IL-10* | NM_001004414.2 | F: CATGCTGCTGGGCCTGAA | Rothwell et al. [4] |
|  |  | R: CGTCTCCTTGATCTGCTTGATG |  |
| *IL-17A* | NM_204460.1 | F: ATGTCTCCGATCCCTTATTCT | Min et al. [5] |
|  |  | R: CCTTTAAGCCTGGTGCTGGAT |  |
| *IFN-γ* | NM_205149 | F: TGATGGCGTGAAGAAGGTG | Bolha et al. [2] |
|  |  | R: GACTGGCTCCTTTTCCTTTTG |  |
| *TGF-β4* | M31160.1 | F: AGGATCTGCAGTGGAGTGGAT | Rothwell et al. [4] |
|  |  | R: CCCCGGGTTGTGTTGGT |  |
| *MIP-1β* | NM_001030360.2 | F: CCCTCATGCTGGTGTTGTGTTCAT | Bolha et al. [2] |
| (*CCL4*) |  | R: CATCCCTGGTGCATCAGTTCAGTT |  |
| *CD25* | NM_204596.1 | F: CGAAGCAAGCAAACAATTCA | Shanmugasundaram et al.[6] |
| (*IL-2Rα*) |  | R: ATGGTGCCAGTGGTAGGAAG |  |
| *CTLA-4* | NM_001040091.1 | F: AAATGGGACGCAACTCTACG | Shanmugasundaram et al. [6] |
|  |  | R: CGACAATGGCTGAGATGATG |  |
| *LAG-3* | XM_025143029.1 | F: CCACTTTGCAGGAGGACACT | Shanmugasundaram et al.[6] |
|  |  | R: GTGACAGCACAGCAATACCG |  |

^a^ TNF = tumour necrosis factor; IL = interleukin; IFN = interferon; TGF = transforming growth factor; MIP = macrophage inflammatory proteins; CCL = C-C motif chemokine ligand; CTLA = T-lymphocyte antigen 4; LAG = lymphocyte activation gene; F = forward primer; R = reverse primer.

1. Li R, Li J, Zhang S, Mi Y, Zhang C. Attenuating effect of melatonin on lipopolysaccharide-induced chicken small intestine inflammation. Poultry Sci. 2018;97(7):2295-302.
2. Bolha L, Benčina D, Cizelj I, Oven I, Slavec B, Rojs OZ, Narat M. Effect of Mycoplasma synoviae and lentogenic Newcastle disease virus coinfection on cytokine and chemokine gene expression in chicken embryos. Poultry Sci. 2013;92(12):3134-43.

3. Liu S, Song M, Yun W, Lee J, Kim H, Cho J. Effect of carvacrol essential oils on immune response and inflammation-related genes expression in broilers challenged by lipopolysaccharide. Poultry Sci. 2019;98(5):2026-33.

4. Rothwell L, Young JR, Zoorob R, Whittaker CA, Hesketh P, Archer A, Smith AL, Kaiser P. Cloning and characterization of chicken IL-10 and its role in the immune response to *Eimeria maxima*. J Immunol. 2004;173(4):2675-82.

5. Min WI, Lillehoj HS. Isolation and characterization of chicken interleukin-17 cDNA. J Interf Cytok Res. 2002;22(11):1123-28.

6. Shanmugasundaram R, Selvaraj RK. Regulatory T cell properties of chicken CD4^+^CD25^+^ cells. J Immunol. 2011;186(4):1997-2002.
